# Supplementary material for: Discovery of coordinately regulated pathways that provide innate protection against interbacterial antagonism
Source: eLife. 2022 Feb 17;11:e74658. doi: 10.7554/eLife.74658 (PMC8926400; doi:10.7554/eLife.74658)
Supplement: Figure 1—source data 1. [file elife-74658-fig1-data1.docx]

Figure 1­Source Data 1. Transposon sequencing-based analysis of *P. aeruginosa* fitness determinants during antagonism by *B. thai* compared to *B. thai* with an inactive T6SS (∆*icmF*)*.*

| Replicate 1 | | | Normalized insertion counts^1^ | |  |
| --- | --- | --- | --- | --- | --- |
| Locus ID^2^ | Gene name | TA sites | + *B.t.* WT | + *B.t*. ∆T6S | Fold change (∆T6S/WT) |
| *Genes with higher insertion frequency + B.t.* ∆T6S | | | | | |
| PA0005 | lptA | 11 | 2.26180694 | 7 | 3.094870688 |
| PA0011 | PA0011 | 22 | 115.352154 | 197 | 1.707813796 |
| PA0066 | PA0066 | 3 | 382.245374 | 651 | 1.703094515 |
| PA0070 | PA0070 | 16 | 236.358826 | 1681 | 7.112067818 |
| PA0071 | PA0071 | 27 | 743.003581 | 4973 | 6.693103677 |
| PA0072 | PA0072 | 9 | 107.43583 | 656 | 6.10597042 |
| PA0073 | PA0073 | 4 | 0 | 43 | N.D.^3^ |
| PA0074 | ppkA | 29 | 848.177604 | 4410 | 5.199382746 |
| PA0075 | pppA | 8 | 33.9271042 | 414 | 12.20263296 |
| PA0077 | icmF1 | 30 | 853.832121 | 2554 | 2.991220331 |
| PA0078 | PA0078 | 10 | 225.049791 | 1000 | 4.443461136 |
| PA0079 | PA0079 | 15 | 152.671969 | 1454 | 9.523686696 |
| PA0081 | fha1 | 6 | 186.599073 | 1526 | 8.177961313 |
| PA0082 | PA0082 | 10 | 118.744865 | 611 | 5.14548566 |
| PA0083 | PA0083 | 5 | 211.478949 | 700 | 3.310022124 |
| PA0084 | PA0084 | 18 | 357.365497 | 2221 | 6.214925668 |
| PA0085 | hcp1 | 5 | 150.410162 | 1082 | 7.193662886 |
| PA0087 | PA0087 | 1 | 229.573405 | 588 | 2.561272287 |
| PA0088 | PA0088 | 20 | 326.831103 | 2040 | 6.241756006 |
| PA0089 | PA0089 | 15 | 286.118578 | 1011 | 3.533500016 |
| PA0090 | clpV1 | 15 | 280.464061 | 2196 | 7.829880207 |
| PA0120 | PA0120 | 3 | 97.2576986 | 77 | 0.791711105 |
| PA0125 | PA0125 | 2 | 45.2361389 | 33 | 0.729505232 |
| PA0171 | PA0171 | 10 | 1.13090347 | 21 | 18.56922413 |
| PA0180 | cttP | 5 | 3.39271042 | 61 | 17.9797249 |
| PA0197 | tonB2 | 2 | 5.65451736 | 26 | 4.598093585 |
| PA0278 | PA0278 | 4 | 110.82854 | 47 | 0.424078491 |
| PA0374 | ftsE | 6 | 62.199691 | 213 | 3.424454311 |
| PA0401 | PA0401 | 9 | 3.39271042 | 12 | 3.536995061 |
| PA0407 | gshB | 10 | 7.9163243 | 35 | 4.421243834 |
| PA0409 | pilH | 3 | 365.281821 | 1121 | 3.068863369 |
| PA0424 | mexR | 2 | 251.060571 | 960 | 3.823778446 |
| PA0512 | PA0512 | 5 | 32.7962007 | 48 | 1.463584164 |
| PA0667 | PA0667 | 30 | 148.148355 | 508 | 3.428995212 |
| PA0674 | vreA | 7 | 203.562625 | 210 | 1.031623561 |
| PA0680 | PA0680 | 3 | 89.3413743 | 68 | 0.76112552 |
| PA0712 | PA0712 | 4 | 7.9163243 | 40 | 5.052850096 |
| PA0714.1 | phrD | 4 | 29.4034903 | 41 | 1.394392284 |
| PA0715 | PA0715 | 61 | 15.8326486 | 5 | 0.315803131 |
| PA0753 | PA0753 | 5 | 16.9635521 | 82 | 4.83389325 |
| PA0762 | algU | 13 | 87.0795673 | 177 | 2.032623789 |
| PA0764 | mucB | 5 | 49.7597528 | 125 | 2.512070357 |
| PA0766 | mucD | 11 | 114.221251 | 592 | 5.182923447 |
| PA0819 | PA0819 | 2 | 127.792092 | 102 | 0.798171455 |
| PA0928 | gacS | 18 | 3.39271042 | 985 | 290.3283446 |
| PA0951a | PA0951a | 1 | 6.78542083 | 22 | 3.242245478 |
| PA0967 | ruvB | 17 | 7.9163243 | 54 | 6.82134763 |
| PA0973 | oprL | 11 | 16.9635521 | 90 | 5.305492592 |
| PA1034 | PA1034 | 2 | 6.78542083 | 73 | 10.75835999 |
| PA1045 | PA1045 | 17 | 6.78542083 | 21 | 3.094870683 |
| PA1103 | PA1103 | 2 | 0 | 38 | N.D. |
| PA1151 | imm2 | 16 | 1.13090347 | 4 | 3.536995072 |
| PA1233 | PA1233 | 5 | 18.0944556 | 80 | 4.42124382 |
| PA1235 | PA1235 | 7 | 81.42505 | 105 | 1.289529451 |
| PA1263 | PA1263 | 8 | 185.468169 | 215 | 1.159228568 |
| PA1276 | cobC | 7 | 123.268478 | 193 | 1.565688188 |
| PA1281 | cobV | 5 | 59.937884 | 98 | 1.635026021 |
| PA1285 | PA1285 | 6 | 13.5708417 | 44 | 3.242245468 |
| PA1294 | rnd | 13 | 10.1781312 | 43 | 4.224744126 |
| PA1298 | PA1298 | 0 | 142.493837 | 194 | 1.361462391 |
| PA1394 | PA1394 | 0 | 12.4399382 | 54 | 4.340857578 |
| PA1404 | PA1404 | 2 | 45.2361389 | 178 | 3.934907009 |
| PA1447 | fliQ | 1 | 24.8798764 | 87 | 3.496801937 |
| PA1503 | PA1503 | 4 | 88.2104708 | 45 | 0.510143519 |
| PA1553 | ccoO1 | 11 | 10.1781312 | 38 | 3.733494809 |
| PA1611 | PA1611 | 23 | 88.2104708 | 995 | 11.27984003 |
| PA1615 | PA1615 | 5 | 12.4399382 | 87 | 6.993603875 |
| PA1629 | PA1629 | 5 | 23.7489729 | 81 | 3.410673815 |
| PA1632 | kdpF | 3 | 2.26180694 | 14 | 6.189741376 |
| PA1652 | PA1652 | 11 | 133.44661 | 146 | 1.094070505 |
| PA1656 | PA1656 | 11 | 98.3886021 | 305 | 3.099952571 |
| PA1696 | pscO | 3 | 5.65451736 | 47 | 8.311938404 |
| PA1697 | PA1697 | 13 | 53.1524632 | 87 | 1.636800907 |
| PA1707 | pcrH | 9 | 184.337266 | 193 | 1.046993938 |
| PA1709 | popD | 1 | 156.064679 | 91 | 0.583091578 |
| PA1710 | exsC | 2 | 221.657081 | 79 | 0.35640639 |
| PA1747 | PA1747 | 1 | 0 | 10 | N.D. |
| PA1766 | PA1766 | 13 | 9.04722778 | 148 | 16.35860217 |
| PA1767 | PA1767 | 15 | 50.8906562 | 319 | 6.26834126 |
| PA1776 | sigX | 12 | 22.6180694 | 25 | 1.10531096 |
| PA1801 | clpP | 12 | 24.8798764 | 20 | 0.803862514 |
| PA1879 | PA1879 | 2 | 2.26180694 | 10 | 4.42124384 |
| PA1957 | PA1957 | 5 | 66.7233048 | 87 | 1.30389225 |
| PA2010 | PA2010 | 6 | 62.199691 | 99 | 1.591647778 |
| PA2051 | PA2051 | 7 | 111.959444 | 113 | 1.009294044 |
| PA2088 | PA2088 | 6 | 109.697637 | 100 | 0.911596665 |
| PA2132 | cupA5 | 9 | 213.740756 | 191 | 0.893605897 |
| PA2143 | PA2143 | 2 | 99.5195055 | 83 | 0.834007359 |
| PA2146 | PA2146 | 1 | 0 | 21 | N.D. |
| PA2159 | PA2159 | 2 | 1.13090347 | 16 | 14.14798029 |
| PA2193 | hcnA | 2 | 1.13090347 | 19 | 16.80072659 |
| PA2236 | pslF | 9 | 82.5559535 | 79 | 0.956926747 |
| PA2241 | pslK | 10 | 2.26180694 | 21 | 9.284612063 |
| PA2242 | pslL | 13 | 21.487166 | 83 | 3.862770921 |
| PA2259 | ptxS | 9 | 160.588293 | 201 | 1.251647902 |
| PA2279 | arsC | 3 | 19.225359 | 70 | 3.641024337 |
| PA2284 | PA2284 | 5 | 40.712525 | 122 | 2.996620819 |
| PA2297 | PA2297 | 3 | 19.225359 | 78 | 4.057141404 |
| PA2321 | PA2321 | 3 | 1.13090347 | 16 | 14.14798029 |
| PA2330 | PA2330 | 3 | 2.26180694 | 18 | 7.958238911 |
| PA2449 | PA2449 | 10 | 218.26437 | 261 | 1.195797555 |
| PA2536 | PA2536 | 11 | 192.25359 | 657 | 3.417361413 |
| PA2540 | PA2540 | 19 | 298.558517 | 915 | 3.064725834 |
| PA2541 | PA2541 | 5 | 10.1781312 | 88 | 8.645987979 |
| PA2558 | PA2558 | 4 | 84.8177604 | 94 | 1.108258454 |
| PA2570 | lecA | 14 | 114.221251 | 112 | 0.980553085 |
| PA2577 | PA2577 | 5 | 2.26180694 | 10 | 4.42124384 |
| PA2586 | gacA | 11 | 0 | 685 | N.D. |
| PA2659 | PA2659 | 5 | 0 | 27 | N.D. |
| PA2724 | PA2724 | 4 | 46.3670424 | 141 | 3.040953071 |
| PA2743 | infC | 12 | 2.26180694 | 7 | 3.094870688 |
| PA2830 | htpX | 4 | 74.6396292 | 124 | 1.661315863 |
| PA2853 | oprI | 3 | 163.981003 | 180 | 1.097688127 |
| PA2882 | PA2882 | 7 | 57.6760771 | 26 | 0.450793489 |
| PA2913 | PA2913 | 11 | 84.8177604 | 106 | 1.249738256 |
| PA2946 | PA2946 | 5 | 80.2941465 | 103 | 1.282783422 |
| PA2963 | PA2963 | 17 | 960.137048 | 2075 | 2.161149811 |
| PA2992 | PA2992 | 2 | 28.2725868 | 91 | 3.21866551 |
| PA3033 | PA3033 | 1 | 28.2725868 | 85 | 3.006445806 |
| PA3051 | PA3051 | 2 | 5.65451736 | 50 | 8.842487664 |
| PA3080 | PA3080 | 7 | 122.137575 | 222 | 1.817622464 |
| PA3094.2 | PA3094.2 | 3 | 2.26180694 | 10 | 4.42124384 |
| PA3110 | PA3110 | 3 | 105.174023 | 107 | 1.017361483 |
| PA3111 | folC | 15 | 5.65451736 | 30 | 5.305492598 |
| PA3142 | PA3142 | 5 | 127.792092 | 160 | 1.252033655 |
| PA3145 | wbpL | 34 | 1.13090347 | 5 | 4.42124384 |
| PA3173 | PA3173 | 11 | 12.4399382 | 48 | 3.858540069 |
| PA3205 | PA3205 | 1 | 5.65451736 | 23 | 4.067544325 |
| PA3209 | PA3209 | 7 | 106.304926 | 85 | 0.799586653 |
| PA3214 | PA3214 | 9 | 153.802872 | 149 | 0.968772547 |
| PA3224 | PA3224 | 3 | 228.442501 | 81 | 0.354575001 |
| PA3265 | PA3265 | 3 | 93.8649882 | 150 | 1.598039939 |
| PA3267 | PA3267 | 22 | 1083.40553 | 2305 | 2.127550521 |
| PA3288 | PA3288 | 3 | 10.1781312 | 38 | 3.733494809 |
| PA3298 | PA3298 | 1 | 38.450718 | 64 | 1.664468268 |
| PA3299 | fadD1 | 19 | 24.8798764 | 280 | 11.2540752 |
| PA3317 | PA3317 | 8 | 211.478949 | 647 | 3.059406163 |
| PA3385 | amrZ | 3 | 22.6180694 | 82 | 3.625419949 |
| PA3432 | PA3432 | 3 | 62.199691 | 49 | 0.787785264 |
| PA3439 | folX | 3 | 67.8542083 | 128 | 1.886397369 |
| PA3574 | nalD | 6 | 54.2833667 | 179 | 3.297511022 |
| PA3574a | PA3574a | 3 | 12.4399382 | 57 | 4.582016332 |
| PA3576 | PA3576 | 5 | 148.148355 | 235 | 1.586247785 |
| PA3596 | PA3596 | 8 | 188.86088 | 170 | 0.900133474 |
| PA3616 | PA3616 | 5 | 61.0687875 | 81 | 1.326373149 |
| PA3634 | PA3634 | 2 | 4.52361389 | 36 | 7.958238894 |
| PA3646 | lpxD | 16 | 11.3090347 | 34 | 3.006445811 |
| PA3649 | PA3649 | 20 | 24.8798764 | 98 | 3.93892632 |
| PA3675 | PA3675 | 4 | 363.020015 | 438 | 1.206545044 |
| PA3738 | xerD | 9 | 10.1781312 | 33 | 3.242245492 |
| PA3767 | PA3767 | 6 | 67.8542083 | 75 | 1.105310958 |
| PA3778 | PA3778 | 6 | 33.9271042 | 57 | 1.680072654 |
| PA3888 | PA3888 | 6 | 222.787984 | 198 | 0.888737339 |
| PA3974 | ladS | 36 | 2758.27357 | 9680 | 3.509441596 |
| PA4020 | mpl | 15 | 1.13090347 | 15 | 13.26373152 |
| PA4045 | PA4045 | 6 | 66.7233048 | 93 | 1.393815853 |
| PA4110 | ampC | 21 | 1177.27051 | 1454 | 1.235060241 |
| PA4269 | rpoC | 49 | 29.4034903 | 60 | 2.040574074 |
| PA4275 | nusG | 9 | 9.04722778 | 38 | 4.200181638 |
| PA4277 | tufB | 23 | 2.26180694 | 13 | 5.747616992 |
| PA4318 | PA4318 | 7 | 41.8434285 | 371 | 8.866386271 |
| PA4319 | PA4319 | 13 | 107.43583 | 1727 | 16.07471176 |
| PA4320 | PA4320 | 19 | 79.163243 | 1374 | 17.35654008 |
| PA4321 | PA4321 | 17 | 135.708417 | 934 | 6.88240288 |
| PA4322 | PA4322 | 6 | 24.8798764 | 301 | 12.09813084 |
| PA4323 | PA4323 | 15 | 30.5343937 | 627 | 20.53422138 |
| PA4351 | PA4351 | 4 | 10.1781312 | 59 | 5.79674194 |
| PA4413 | ftsW | 12 | 10.1781312 | 30 | 2.947495902 |
| PA4417 | murE | 16 | 58.8069805 | 34 | 0.578162655 |
| PA4422 | PA4422 | 6 | 0 | 16 | N.D. |
| PA4423 | PA4423 | 18 | 41.8434285 | 127 | 3.035124141 |
| PA4424 | PA4424 | 5 | 67.8542083 | 114 | 1.680072657 |
| PA4428 | sspA | 12 | 184.337266 | 212 | 1.15006588 |
| PA4431 | PA4431 | 8 | 2.26180694 | 7 | 3.094870688 |
| PA4451 | PA4451 | 2 | 5.65451736 | 20 | 3.536995065 |
| PA4610 | PA4610 | 1 | 19.225359 | 69 | 3.589009703 |
| PA4634 | PA4634 | 8 | 0 | 2 | N.D. |
| PA4664 | hemK | 11 | 0 | 14 | N.D. |
| PA4671 | PA4671 | 7 | 2.26180694 | 11 | 4.863368224 |
| PA4726.1 | PA4726.1 | 4 | 1.13090347 | 18 | 15.91647782 |
| PA4729 | panB | 5 | 1.13090347 | 14 | 12.37948275 |
| PA4793 | PA4793 | 9 | 158.326486 | 224 | 1.414798027 |
| PA4795 | PA4795 | 0 | 49.7597528 | 98 | 1.96946316 |
| PA4853 | fis | 4 | 1.13090347 | 13 | 11.49523398 |
| PA4890 | desT | 2 | 0 | 25 | N.D. |
| PA4932 | rplI | 4 | 11.3090347 | 10 | 0.884248768 |
| PA4952 | PA4952 | 5 | 4.52361389 | 15 | 3.315932872 |
| PA4960 | PA4960 | 11 | 36.1889111 | 59 | 1.630333663 |
| PA5000 | wapR | 21 | 6.78542083 | 25 | 3.684369861 |
| PA5005 | PA5005 | 32 | 10.1781312 | 17 | 1.670247678 |
| PA5016 | aceF | 7 | 3.39271042 | 20 | 5.894991769 |
| PA5049 | rpmE | 7 | 4.52361389 | 17 | 3.758057255 |
| PA5060 | phaF | 4 | 176.420942 | 129 | 0.731205709 |
| PA5113 | PA5113 | 15 | 223.918887 | 707 | 3.157393329 |
| PA5114 | PA5114 | 37 | 246.536957 | 3226 | 13.08525926 |
| PA5162 | rmlD | 11 | 6.78542083 | 53 | 7.810864105 |
| PA5239 | rho | 10 | 57.6760771 | 161 | 2.791451987 |
| PA5241 | ppx | 15 | 11.3090347 | 35 | 3.094870688 |
| PA5259 | hemD | 13 | 27.1416833 | 121 | 4.458087535 |
| PA5280 | sss | 8 | 5.65451736 | 17 | 3.006445806 |
| PA5285 | PA5285 | 1 | 9.04722778 | 79 | 8.731956564 |
| PA5366 | pstB | 11 | 1.13090347 | 9 | 7.958238911 |
| PA5405 | PA5405 | 3 | 105.174023 | 59 | 0.560975023 |
| PA5409 | PA5409 | 2 | 36.1889111 | 32 | 0.884248766 |
| PA5555 | atpG | 15 | 1.13090347 | 6 | 5.305492608 |
|  |  |  |  |  |  |
| *Genes with higher insertion frequency + B.t.* WT | | | | | |
| PA0285 | PA0285 | 25 | 3788.52663 | 975 | 0.257355984 |
| PA0427 | oprM | 21 | 27.1416833 | 7 | 0.25790589 |
| PA0542 | PA0542 | 0 | 83.6868569 | 86 | 1.027640459 |
| PA0624 | PA0624 | 4 | 143.624741 | 117 | 0.814622879 |
| PA0635 | PA0635 | 4 | 37.3198146 | 36 | 0.964635017 |
| PA0787 | PA0787 | 5 | 106.304926 | 148 | 1.392221467 |
| PA0823 | PA0823 | 0 | 44.1052354 | 13 | 0.294749589 |
| PA0905 | rsmA | 4 | 166.24281 | 42 | 0.252642505 |
| PA1030.1 | PA1030.1 | 2 | 62.199691 | 13 | 0.209004254 |
| PA1082 | flgG | 6 | 1143.34341 | 559 | 0.488916974 |
| PA1117 | PA1117 | 1 | 53.1524632 | 13 | 0.244579446 |
| PA1149 | PA1149 | 3 | 97.2576986 | 30 | 0.308458872 |
| PA1159 | PA1159 | 3 | 36.1889111 | 11 | 0.303960513 |
| PA1300 | PA1300 | 2 | 94.9958916 | 190 | 2.000086496 |
| PA1444 | fliN | 4 | 220.526177 | 97 | 0.439857079 |
| PA1612 | PA1612 | 9 | 534.917342 | 153 | 0.2860255 |
| PA1635 | kdpC | 7 | 56.5451736 | 115 | 2.033772163 |
| PA1644 | PA1644 | 7 | 94.9958916 | 67 | 0.705293659 |
| PA1692 | PA1692 | 4 | 45.2361389 | 13 | 0.287380849 |
| PA1718 | pscE | 0 | 134.577513 | 91 | 0.676190234 |
| PA1891 | PA1891 | 4 | 47.4979458 | 57 | 1.200051898 |
| PA2008 | fahA | 11 | 196.777204 | 47 | 0.238848805 |
| PA2066 | PA2066 | 5 | 57.6760771 | 59 | 1.022954455 |
| PA2145 | PA2145 | 5 | 59.937884 | 66 | 1.101139974 |
| PA2161 | PA2161 | 2 | 23.7489729 | 7 | 0.294749589 |
| PA2198 | PA2198 | 5 | 228.442501 | 146 | 0.639110495 |
| PA2312 | PA2312 | 7 | 121.006672 | 94 | 0.77681667 |
| PA2315 | PA2315 | 10 | 99.5195055 | 194 | 1.949366599 |
| PA2334 | PA2334 | 7 | 16.9635521 | 39 | 2.29904679 |
| PA2351 | PA2351 | 9 | 122.137575 | 107 | 0.876061278 |
| PA2469 | PA2469 | 6 | 195.646301 | 208 | 1.063143024 |
| PA2492 | mexT | 8 | 20109.7255 | 5718 | 0.284340032 |
| PA2495 | oprN | 10 | 107.43583 | 104 | 0.968019701 |
| PA2497 | PA2497 | 5 | 41.8434285 | 49 | 1.171032149 |
| PA2614 | lolA | 5 | 71.2469187 | 7 | 0.098249863 |
| PA2970 | rpmF | 5 | 33.9271042 | 7 | 0.206324712 |
| PA3017 | PA3017 | 3 | 58.8069805 | 54 | 0.918258335 |
| PA3085 | PA3085 | 2 | 4.52361389 | 4 | 0.884248766 |
| PA3139.1 | PA3139.1 | 3 | 19.225359 | 3 | 0.1560439 |
| PA3181 | PA3181 | 5 | 72.3778222 | 22 | 0.303960513 |
| PA3194 | edd | 9 | 12.4399382 | 17 | 1.366566274 |
| PA3411 | PA3411 | 2 | 148.148355 | 39 | 0.263249632 |
| PA3448 | PA3448 | 8 | 187.729976 | 140 | 0.745751973 |
| PA3480 | PA3480 | 10 | 30.5343937 | 25 | 0.818748859 |
| PA3520 | PA3520 | 4 | 63.3305944 | 20 | 0.315803131 |
| PA3808 | PA3808 | 2 | 52.0215597 | 2 | 0.038445599 |
| PA3813 | iscU | 3 | 44.1052354 | 8 | 0.181384362 |
| PA3816 | cysE | 8 | 144.755644 | 27 | 0.186521225 |
| PA3833 | PA3833 | 2 | 24.8798764 | 41 | 1.647918154 |
| PA3844 | PA3844 | 8 | 1015.55132 | 316 | 0.311161035 |
| PA3937 | PA3937 | 4 | 33.9271042 | 2 | 0.058949918 |
| PA4033 | PA4033 | 3 | 12.4399382 | 1 | 0.080386251 |
| PA4092 | hpaC | 1 | 42.9743319 | 6 | 0.139618226 |
| PA4138 | tyrS | 10 | 934.126268 | 267 | 0.285828596 |
| PA4479 | mreD | 6 | 63.3305944 | 20 | 0.315803131 |
| PA4482 | gatC | 2 | 29.4034903 | 4 | 0.136038272 |
| PA4561 | ribF | 7 | 30.5343937 | 6 | 0.196499726 |
| PA4637 | PA4637 | 1 | 91.6031812 | 29 | 0.316582892 |
| PA4663 | moeB | 9 | 31.6652972 | 7 | 0.221062192 |
| PA4732 | pgi | 24 | 85.9486639 | 121 | 1.407817114 |
| PA4822 | PA4822 | 9 | 27.1416833 | 51 | 1.87902863 |
| PA4856 | retS | 20 | 669.494855 | 108 | 0.161315653 |
| PA4916 | PA4916 | 5 | 54.2833667 | 18 | 0.331593287 |
| PA4918 | PA4918 | 10 | 464.801327 | 100 | 0.215145685 |
| PA5062 | PA5062 | 0 | 23.7489729 | 2 | 0.084214168 |
| PA5134 | PA5134 | 11 | 360.758208 | 187 | 0.518352724 |
| PA5161 | rmlB | 19 | 49.7597528 | 2 | 0.040193126 |
| PA5183a | PA5183a | 3 | 102.912216 | 125 | 1.214627426 |
| PA5203 | gshA | 25 | 1049.47842 | 383 | 0.364943188 |
| PA5231 | PA5231 | 27 | 4334.75301 | 4400 | 1.015052066 |
| PA5443 | uvrD | 22 | 67.8542083 | 47 | 0.692661534 |

| **Replicate 2** | | | **Normalized insertion counts** | |  |
| --- | --- | --- | --- | --- | --- |
| **Locus ID** | **Gene name** | **TA sites** | **+ *B.t*. WT** | **+ *B.t*. ∆T6S** | **Fold change (∆T6S /WT)** |
| *Genes with higher insertion frequency + B.t.* ∆T6S | | | | | |
| PA0005 | lptA | 11 | 25.2832219 | 7 | 0.276863449 |
| PA0011 | PA0011 | 22 | 5.41783325 | 20 | 3.691512652 |
| PA0066 | PA0066 | 3 | 12.6416109 | 51 | 4.034295969 |
| PA0070 | PA0070 | 16 | 21.671333 | 156 | 7.198449671 |
| PA0071 | PA0071 | 27 | 46.9545549 | 329 | 7.006774975 |
| PA0072 | PA0072 | 9 | 5.41783325 | 39 | 7.198449671 |
| PA0073 | PA0073 | 4 | 1.80594442 | 6 | 3.322361386 |
| PA0074 | ppkA | 29 | 68.6258879 | 300 | 4.37152814 |
| PA0075 | pppA | 8 | 9.02972209 | 51 | 5.648014357 |
| PA0077 | icmF1 | 30 | 25.2832219 | 187 | 7.396209277 |
| PA0078 | PA0078 | 10 | 19.8653886 | 65 | 3.272022578 |
| PA0079 | PA0079 | 15 | 12.6416109 | 100 | 7.910384253 |
| PA0081 | fha1 | 6 | 21.671333 | 117 | 5.398837253 |
| PA0082 | PA0082 | 10 | 7.22377767 | 49 | 6.783154497 |
| PA0083 | PA0083 | 5 | 5.41783325 | 48 | 8.859630364 |
| PA0084 | PA0084 | 18 | 25.2832219 | 153 | 6.051443954 |
| PA0085 | hcp1 | 5 | 16.2534998 | 71 | 4.368289971 |
| PA0087 | PA0087 | 1 | 7.22377767 | 32 | 4.429815182 |
| PA0088 | PA0088 | 20 | 25.2832219 | 188 | 7.435761198 |
| PA0089 | PA0089 | 15 | 41.5367216 | 85 | 2.046382013 |
| PA0090 | clpV1 | 15 | 10.8356665 | 131 | 12.08970393 |
| PA0120 | PA0120 | 3 | 1.80594442 | 7 | 3.876088284 |
| PA0125 | PA0125 | 2 | 1.80594442 | 6 | 3.322361386 |
| PA0171 | PA0171 | 10 | 5.41783325 | 3 | 0.553726898 |
| PA0180 | cttP | 5 | 1.80594442 | 3 | 1.661180693 |
| PA0197 | tonB2 | 2 | 1.80594442 | 3 | 1.661180693 |
| PA0278 | PA0278 | 4 | 1.80594442 | 7 | 3.876088284 |
| PA0374 | ftsE | 6 | 5.41783325 | 18 | 3.322361386 |
| PA0401 | PA0401 | 9 | 32.5069995 | 44 | 1.353554639 |
| PA0407 | gshB | 10 | 16.2534998 | 14 | 0.861352952 |
| PA0409 | pilH | 3 | 50.5664437 | 89 | 1.760060496 |
| PA0424 | mexR | 2 | 23.4772774 | 53 | 2.257501968 |
| PA0512 | PA0512 | 5 | 1.80594442 | 7 | 3.876088284 |
| PA0667 | PA0667 | 30 | 7.22377767 | 11 | 1.522748969 |
| PA0674 | vreA | 7 | 5.41783325 | 18 | 3.322361386 |
| PA0680 | PA0680 | 3 | 5.41783325 | 18 | 3.322361386 |
| PA0712 | PA0712 | 4 | 1.80594442 | 3 | 1.661180693 |
| PA0714.1 | phrD | 4 | 1.80594442 | 7 | 3.876088284 |
| PA0715 | PA0715 | 61 | 1.80594442 | 14 | 7.752176568 |
| PA0753 | PA0753 | 5 | 7.22377767 | 7 | 0.969022071 |
| PA0762 | algU | 13 | 1.80594442 | 16 | 8.859630364 |
| PA0764 | mucB | 5 | 1.80594442 | 6 | 3.322361386 |
| PA0766 | mucD | 11 | 16.2534998 | 22 | 1.353554639 |
| PA0819 | PA0819 | 2 | 5.41783325 | 17 | 3.137785754 |
| PA0928 | gacS | 18 | 7.22377767 | 50 | 6.921586222 |
| PA0951a | PA0951a | 1 | 0.1 | 2 | 20 |
| PA0967 | ruvB | 17 | 10.8356665 | 5 | 0.461439081 |
| PA0973 | oprL | 11 | 3.61188884 | 8 | 2.214907591 |
| PA1034 | PA1034 | 2 | 3.61188884 | 0 | 0 |
| PA1045 | PA1045 | 17 | 3.61188884 | 4 | 1.107453795 |
| PA1103 | PA1103 | 2 | 1.80594442 | 2 | 1.107453795 |
| PA1151 | imm2 | 16 | 0 | 0 | N.D. |
| PA1233 | PA1233 | 5 | 5.41783325 | 8 | 1.476605061 |
| PA1235 | PA1235 | 7 | 1.80594442 | 13 | 7.198449671 |
| PA1263 | PA1263 | 8 | 5.41783325 | 20 | 3.691512652 |
| PA1276 | cobC | 7 | 1.80594442 | 15 | 8.305903466 |
| PA1281 | cobV | 5 | 1.80594442 | 6 | 3.322361386 |
| PA1285 | PA1285 | 6 | 3.61188884 | 4 | 1.107453795 |
| PA1294 | rnd | 13 | 5.41783325 | 6 | 1.107453795 |
| PA1298 | PA1298 | 0 | 5.41783325 | 17 | 3.137785754 |
| PA1394 | PA1394 | 0 | 1.80594442 | 4 | 2.214907591 |
| PA1404 | PA1404 | 2 | 14.4475553 | 11 | 0.761374484 |
| PA1447 | fliQ | 1 | 3.61188884 | 1 | 0.276863449 |
| PA1503 | PA1503 | 4 | 1.80594442 | 11 | 6.090995875 |
| PA1553 | ccoO1 | 11 | 3.61188884 | 9 | 2.49177104 |
| PA1611 | PA1611 | 23 | 61.4021102 | 111 | 1.80775546 |
| PA1615 | PA1615 | 5 | 5.41783325 | 2 | 0.369151265 |
| PA1629 | PA1629 | 5 | 16.2534998 | 6 | 0.369151265 |
| PA1632 | kdpF | 3 | 0 | 0 | N.D. |
| PA1652 | PA1652 | 11 | 1.80594442 | 14 | 7.752176568 |
| PA1656 | PA1656 | 11 | 19.8653886 | 33 | 1.661180693 |
| PA1696 | pscO | 3 | 5.41783325 | 7 | 1.292029428 |
| PA1697 | PA1697 | 13 | 3.61188884 | 11 | 3.045497938 |
| PA1707 | pcrH | 9 | 5.41783325 | 22 | 4.060663917 |
| PA1709 | popD | 1 | 1.80594442 | 8 | 4.429815182 |
| PA1710 | exsC | 2 | 5.41783325 | 17 | 3.137785754 |
| PA1747 | PA1747 | 1 | 1.80594442 | 2 | 1.107453795 |
| PA1766 | PA1766 | 13 | 1.80594442 | 5 | 2.768634489 |
| PA1767 | PA1767 | 15 | 19.8653886 | 8 | 0.402710471 |
| PA1776 | sigX | 12 | 1.80594442 | 8 | 4.429815182 |
| PA1801 | clpP | 12 | 3.61188884 | 11 | 3.045497938 |
| PA1879 | PA1879 | 2 | 0 | 0 | N.D. |
| PA1957 | PA1957 | 5 | 1.80594442 | 10 | 5.537268977 |
| PA2010 | PA2010 | 6 | 3.61188884 | 13 | 3.599224835 |
| PA2051 | PA2051 | 7 | 3.61188884 | 13 | 3.599224835 |
| PA2088 | PA2088 | 6 | 1.80594442 | 7 | 3.876088284 |
| PA2132 | cupA5 | 9 | 7.22377767 | 23 | 3.183929662 |
| PA2143 | PA2143 | 2 | 1.80594442 | 7 | 3.876088284 |
| PA2146 | PA2146 | 1 | 1.80594442 | 1 | 0.553726898 |
| PA2159 | PA2159 | 2 | 0 | 0 | #DIV/0! |
| PA2193 | hcnA | 2 | 0 | 0 | N.D. |
| PA2236 | pslF | 9 | 3.61188884 | 14 | 3.876088284 |
| PA2241 | pslK | 10 | 0 | 0 | N.D. |
| PA2242 | pslL | 13 | 1.80594442 | 1 | 0.553726898 |
| PA2259 | ptxS | 9 | 1.80594442 | 14 | 7.752176568 |
| PA2279 | arsC | 3 | 0.1 | 5 | 50 |
| PA2284 | PA2284 | 5 | 3.61188884 | 7 | 1.938044142 |
| PA2297 | PA2297 | 3 | 7.22377767 | 10 | 1.384317244 |
| PA2321 | PA2321 | 3 | 0 | 0 | N.D. |
| PA2330 | PA2330 | 3 | 0.1 | 1 | 10 |
| PA2449 | PA2449 | 10 | 7.22377767 | 26 | 3.599224835 |
| PA2536 | PA2536 | 11 | 16.2534998 | 96 | 5.906420243 |
| PA2540 | PA2540 | 19 | 48.7604993 | 107 | 2.194399187 |
| PA2541 | PA2541 | 5 | 0.1 | 16 | 160 |
| PA2558 | PA2558 | 4 | 1.80594442 | 6 | 3.322361386 |
| PA2570 | lecA | 14 | 3.61188884 | 11 | 3.045497938 |
| PA2577 | PA2577 | 5 | 0 | 0 | N.D. |
| PA2586 | gacA | 11 | 3.61188884 | 35 | 9.69022071 |
| PA2659 | PA2659 | 5 | 0.1 | 2 | 20 |
| PA2724 | PA2724 | 4 | 12.6416109 | 21 | 1.661180693 |
| PA2743 | infC | 12 | 1.80594442 | 1 | 0.553726898 |
| PA2830 | htpX | 4 | 5.41783325 | 22 | 4.060663917 |
| PA2853 | oprI | 3 | 9.02972209 | 30 | 3.322361386 |
| PA2882 | PA2882 | 7 | 1.80594442 | 8 | 4.429815182 |
| PA2913 | PA2913 | 11 | 1.80594442 | 12 | 6.644722773 |
| PA2946 | PA2946 | 5 | 3.61188884 | 12 | 3.322361386 |
| PA2963 | PA2963 | 17 | 28.8951107 | 118 | 4.083735871 |
| PA2992 | PA2992 | 2 | 9.02972209 | 11 | 1.218199175 |
| PA3033 | PA3033 | 1 | 7.22377767 | 5 | 0.692158622 |
| PA3051 | PA3051 | 2 | 1.80594442 | 6 | 3.322361386 |
| PA3080 | PA3080 | 7 | 3.61188884 | 13 | 3.599224835 |
| PA3094.2 | PA3094.2 | 3 | 1.80594442 | 0 | 0 |
| PA3110 | PA3110 | 3 | 5.41783325 | 20 | 3.691512652 |
| PA3111 | folC | 15 | 1.80594442 | 5 | 2.768634489 |
| PA3142 | PA3142 | 5 | 5.41783325 | 18 | 3.322361386 |
| PA3145 | wbpL | 34 | 0 | 0 | N.D. |
| PA3173 | PA3173 | 11 | 1.80594442 | 2 | 1.107453795 |
| PA3205 | PA3205 | 1 | 1.80594442 | 0 | 0 |
| PA3209 | PA3209 | 7 | 7.22377767 | 28 | 3.876088284 |
| PA3214 | PA3214 | 9 | 3.61188884 | 11 | 3.045497938 |
| PA3224 | PA3224 | 3 | 1.80594442 | 12 | 6.644722773 |
| PA3265 | PA3265 | 3 | 3.61188884 | 11 | 3.045497938 |
| PA3267 | PA3267 | 22 | 41.5367216 | 248 | 5.970620463 |
| PA3288 | PA3288 | 3 | 1.80594442 | 6 | 3.322361386 |
| PA3298 | PA3298 | 1 | 1.80594442 | 8 | 4.429815182 |
| PA3299 | fadD1 | 19 | 1.80594442 | 7 | 3.876088284 |
| PA3317 | PA3317 | 8 | 46.9545549 | 64 | 1.363020056 |
| PA3385 | amrZ | 3 | 3.61188884 | 5 | 1.384317244 |
| PA3432 | PA3432 | 3 | 1.80594442 | 14 | 7.752176568 |
| PA3439 | folX | 3 | 1.80594442 | 7 | 3.876088284 |
| PA3574 | nalD | 6 | 1.80594442 | 14 | 7.752176568 |
| PA3574a | PA3574a | 3 | 3.61188884 | 4 | 1.107453795 |
| PA3576 | PA3576 | 5 | 7.22377767 | 24 | 3.322361386 |
| PA3596 | PA3596 | 8 | 5.41783325 | 18 | 3.322361386 |
| PA3616 | PA3616 | 5 | 1.80594442 | 7 | 3.876088284 |
| PA3634 | PA3634 | 2 | 0.1 | 6 | 60 |
| PA3646 | lpxD | 16 | 1.80594442 | 1 | 0.553726898 |
| PA3649 | PA3649 | 20 | 10.8356665 | 13 | 1.199741612 |
| PA3675 | PA3675 | 4 | 16.2534998 | 50 | 3.076260543 |
| PA3738 | xerD | 9 | 9.02972209 | 8 | 0.885963036 |
| PA3767 | PA3767 | 6 | 1.80594442 | 9 | 4.98354208 |
| PA3778 | PA3778 | 6 | 1.80594442 | 7 | 3.876088284 |
| PA3888 | PA3888 | 6 | 5.41783325 | 19 | 3.506937019 |
| PA3974 | ladS | 36 | 574.290325 | 842 | 1.466157383 |
| PA4020 | mpl | 15 | 1.80594442 | 3 | 1.661180693 |
| PA4045 | PA4045 | 6 | 1.80594442 | 10 | 5.537268977 |
| PA4110 | ampC | 21 | 37.9248328 | 125 | 3.295993439 |
| PA4269 | rpoC | 49 | 1.80594442 | 8 | 4.429815182 |
| PA4275 | nusG | 9 | 3.61188884 | 3 | 0.830590347 |
| PA4277 | tufB | 23 | 1.80594442 | 6 | 3.322361386 |
| PA4318 | PA4318 | 7 | 10.8356665 | 69 | 6.367859324 |
| PA4319 | PA4319 | 13 | 21.671333 | 186 | 8.582766915 |
| PA4320 | PA4320 | 19 | 16.2534998 | 160 | 9.844033738 |
| PA4321 | PA4321 | 17 | 12.6416109 | 86 | 6.802930458 |
| PA4322 | PA4322 | 6 | 10.8356665 | 22 | 2.030331958 |
| PA4323 | PA4323 | 15 | 12.6416109 | 63 | 4.98354208 |
| PA4351 | PA4351 | 4 | 9.02972209 | 4 | 0.442981518 |
| PA4413 | ftsW | 12 | 1.80594442 | 9 | 4.98354208 |
| PA4417 | murE | 16 | 1.80594442 | 12 | 6.644722773 |
| PA4422 | PA4422 | 6 | 1.80594442 | 1 | 0.553726898 |
| PA4423 | PA4423 | 18 | 12.6416109 | 14 | 1.107453795 |
| PA4424 | PA4424 | 5 | 5.41783325 | 20 | 3.691512652 |
| PA4428 | sspA | 12 | 7.22377767 | 26 | 3.599224835 |
| PA4431 | PA4431 | 8 | 0.1 | 3 | 30 |
| PA4451 | PA4451 | 2 | 0.1 | 1 | 10 |
| PA4610 | PA4610 | 1 | 5.41783325 | 5 | 0.922878163 |
| PA4634 | PA4634 | 8 | 3.61188884 | 17 | 4.706678631 |
| PA4664 | hemK | 11 | 5.41783325 | 3 | 0.553726898 |
| PA4671 | PA4671 | 7 | 14.4475553 | 14 | 0.969022071 |
| PA4726.1 | PA4726.1 | 4 | 1.80594442 | 3 | 1.661180693 |
| PA4729 | panB | 5 | 1.80594442 | 13 | 7.198449671 |
| PA4793 | PA4793 | 9 | 3.61188884 | 19 | 5.260405528 |
| PA4795 | PA4795 | 0 | 1.80594442 | 7 | 3.876088284 |
| PA4853 | fis | 4 | 0.1 | 1 | 10 |
| PA4890 | desT | 2 | 0.1 | 1 | 10 |
| PA4932 | rplI | 4 | 1.80594442 | 7 | 3.876088284 |
| PA4952 | PA4952 | 5 | 1.80594442 | 3 | 1.661180693 |
| PA4960 | PA4960 | 11 | 3.61188884 | 11 | 3.045497938 |
| PA5000 | wapR | 21 | 3.61188884 | 2 | 0.553726898 |
| PA5005 | PA5005 | 32 | 1.80594442 | 17 | 9.413357262 |
| PA5016 | aceF | 7 | 5.41783325 | 7 | 1.292029428 |
| PA5049 | rpmE | 7 | 18.0594442 | 18 | 0.996708416 |
| PA5060 | phaF | 4 | 5.41783325 | 24 | 4.429815182 |
| PA5113 | PA5113 | 15 | 21.671333 | 62 | 2.860922305 |
| PA5114 | PA5114 | 37 | 61.4021102 | 320 | 5.211547273 |
| PA5162 | rmlD | 11 | 1.80594442 | 6 | 3.322361386 |
| PA5239 | rho | 10 | 5.41783325 | 20 | 3.691512652 |
| PA5241 | ppx | 15 | 16.2534998 | 18 | 1.107453795 |
| PA5259 | hemD | 13 | 3.61188884 | 5 | 1.384317244 |
| PA5280 | sss | 8 | 3.61188884 | 4 | 1.107453795 |
| PA5285 | PA5285 | 1 | 1.80594442 | 5 | 2.768634489 |
| PA5366 | pstB | 11 | 3.61188884 | 14 | 3.876088284 |
| PA5405 | PA5405 | 3 | 1.80594442 | 10 | 5.537268977 |
| PA5409 | PA5409 | 2 | 1.80594442 | 7 | 3.876088284 |
| PA5555 | atpG | 15 | 0.1 | 2 | 20 |
|  |  |  |  |  |  |
| *Genes with higher insertion frequency + B.t.* WT | | | | | |
| PA0285 | PA0285 | 25 | 270.8916627 | 151 | 0.55741841 |
| PA0427 | oprM | 21 | 0.1 | 3 | 30 |
| PA0542 | PA0542 | 0 | 14.44755534 | 4 | 0.276863449 |
| PA0624 | PA0624 | 4 | 25.28322185 | 8 | 0.31641537 |
| PA0635 | PA0635 | 4 | 16.25349976 | 4 | 0.246100843 |
| PA0787 | PA0787 | 5 | 21.67133301 | 6 | 0.276863449 |
| PA0823 | PA0823 | 0 | 1.805944418 | 1 | 0.553726898 |
| PA0905 | rsmA | 4 | 30.7010551 | 10 | 0.325721705 |
| PA1030.1 | PA1030.1 | 2 | 0 | 0 | N.D. |
| PA1082 | flgG | 6 | 83.07344322 | 21 | 0.252788366 |
| PA1117 | PA1117 | 1 | 0.1 | 1 | 10 |
| PA1149 | PA1149 | 3 | 7.223777672 | 5 | 0.692158622 |
| PA1159 | PA1159 | 3 | 0.1 | 4 | 40 |
| PA1300 | PA1300 | 2 | 21.67133301 | 7 | 0.323007357 |
| PA1444 | fliN | 4 | 12.64161093 | 4 | 0.31641537 |
| PA1612 | PA1612 | 9 | 50.5664437 | 33 | 0.652606701 |
| PA1635 | kdpC | 7 | 19.8653886 | 5 | 0.251694044 |
| PA1644 | PA1644 | 7 | 14.44755534 | 4 | 0.276863449 |
| PA1692 | PA1692 | 4 | 3.611888836 | 3 | 0.830590347 |
| PA1718 | pscE | 0 | 16.25349976 | 4 | 0.246100843 |
| PA1891 | PA1891 | 4 | 14.44755534 | 3 | 0.207647587 |
| PA2008 | fahA | 11 | 7.223777672 | 8 | 1.107453795 |
| PA2066 | PA2066 | 5 | 10.83566651 | 3 | 0.276863449 |
| PA2145 | PA2145 | 5 | 12.64161093 | 4 | 0.31641537 |
| PA2161 | PA2161 | 2 | 0.1 | 4 | 40 |
| PA2198 | PA2198 | 5 | 41.53672161 | 9 | 0.216675743 |
| PA2312 | PA2312 | 7 | 21.67133301 | 7 | 0.323007357 |
| PA2315 | PA2315 | 10 | 16.25349976 | 4 | 0.246100843 |
| PA2334 | PA2334 | 7 | 0.1 | 13 | 130 |
| PA2351 | PA2351 | 9 | 19.8653886 | 6 | 0.302032853 |
| PA2469 | PA2469 | 6 | 34.31294394 | 11 | 0.32057873 |
| PA2492 | mexT | 8 | 2248.4008 | 677 | 0.301102899 |
| PA2495 | oprN | 10 | 19.8653886 | 6 | 0.302032853 |
| PA2497 | PA2497 | 5 | 10.83566651 | 1 | 0.092287816 |
| PA2614 | lolA | 5 | 1.805944418 | 1 | 0.553726898 |
| PA2970 | rpmF | 5 | 3.611888836 | 9 | 2.49177104 |
| PA3017 | PA3017 | 3 | 12.64161093 | 3 | 0.237311528 |
| PA3085 | PA3085 | 2 | 10.83566651 | 2 | 0.184575633 |
| PA3139.1 | PA3139.1 | 3 | 0 | 0 | N.D. |
| PA3181 | PA3181 | 5 | 1.805944418 | 3 | 1.661180693 |
| PA3194 | edd | 9 | 14.44755534 | 4 | 0.276863449 |
| PA3411 | PA3411 | 2 | 9.029722089 | 5 | 0.553726898 |
| PA3448 | PA3448 | 8 | 10.83566651 | 3 | 0.276863449 |
| PA3480 | PA3480 | 10 | 27.08916627 | 9 | 0.332236139 |
| PA3520 | PA3520 | 4 | 3.611888836 | 7 | 1.938044142 |
| PA3808 | PA3808 | 2 | 0.1 | 5 | 50 |
| PA3813 | iscU | 3 | 5.417833254 | 2 | 0.369151265 |
| PA3816 | cysE | 8 | 10.83566651 | 7 | 0.646014714 |
| PA3833 | PA3833 | 2 | 16.25349976 | 3 | 0.184575633 |
| PA3844 | PA3844 | 8 | 54.17833254 | 24 | 0.442981518 |
| PA3937 | PA3937 | 4 | 3.611888836 | 0 | 0 |
| PA4033 | PA4033 | 3 | 3.611888836 | 0 | 0 |
| PA4092 | hpaC | 1 | 0 | 0 | N.D. |
| PA4138 | tyrS | 10 | 28.89511069 | 14 | 0.484511036 |
| PA4479 | mreD | 6 | 3.611888836 | 2 | 0.553726898 |
| PA4482 | gatC | 2 | 0.1 | 1 | 10 |
| PA4561 | ribF | 7 | 3.611888836 | 0 | 0 |
| PA4637 | PA4637 | 1 | 7.223777672 | 3 | 0.415295173 |
| PA4663 | moeB | 9 | 0.1 | 1 | 10 |
| PA4732 | pgi | 24 | 1.805944418 | 21 | 11.62826485 |
| PA4822 | PA4822 | 9 | 10.83566651 | 1 | 0.092287816 |
| PA4856 | retS | 20 | 99.32694298 | 29 | 0.291965092 |
| PA4916 | PA4916 | 5 | 28.89511069 | 6 | 0.207647587 |
| PA4918 | PA4918 | 10 | 27.08916627 | 27 | 0.996708416 |
| PA5062 | PA5062 | 0 | 0 | 0 | N.D. |
| PA5134 | PA5134 | 11 | 27.08916627 | 5 | 0.184575633 |
| PA5161 | rmlB | 19 | 0 | 0 | N.D. |
| PA5183a | PA5183a | 3 | 12.64161093 | 3 | 0.237311528 |
| PA5203 | gshA | 25 | 2293.549411 | 700 | 0.305203802 |
| PA5231 | PA5231 | 27 | 12.64161093 | 3 | 0.237311528 |
| PA5443 | uvrD | 22 | 14.44755534 | 4 | 0.276863449 |

1. Insertion counts are normalized by the total sequencing reads within each sample.
2. Genes with 3-fold more or greater difference in normalized insertion frequency in at least one of two replicate experiments and with at least 10 normalized insertions from one library in that experiment are included. Genes meeting these criteria in both replicates are highlighted in grey.
3. The gene was not detected in the wild-type sample.
