# Supplementary material for: Discovery of coordinately regulated pathways that provide innate protection against interbacterial antagonism
Source: eLife. 2022 Feb 17;11:e74658. doi: 10.7554/eLife.74658 (PMC8926400; doi:10.7554/eLife.74658)
Supplement: Figure 2—source data 1. [file elife-74658-fig2-data1.docx]

Figure 2­Source Data 1. Transposon sequencing-based analysis of *P. aeruginosa* fitness determinants during antagonism by *B. thai* compared to *B. thai tle3*^S264A^*.*

|  | | | Normalized insertion counts | |  |
| --- | --- | --- | --- | --- | --- |
| Locus ID | Gene name | TA sites | + *B.t.* WT | + *B.t*. *tle3*^S264A^ | Fold change (*tle3*^S264A^/WT) |
| *Genes with higher insertion frequency + B.t. tle3^S264A^* | | | | | |
| PA0005 | lptA | 11 | 2 | 17.00958 | 8.504789999 |
| PA0073 | PA0073 | 4 | 0.1 | 34.01916 | 340.1916 |
| PA0075 | pppA | 8 | 30 | 100.5111545 | 3.350371818 |
| PA0079 | PA0079 | 15 | 135 | 415.9615472 | 3.081196646 |
| PA0171 | PA0171 | 10 | 1 | 7.731627272 | 7.731627272 |
| PA0180 | cttP | 5 | 3 | 52.5750654 | 17.5250218 |
| PA0197 | tonB2 | 2 | 5 | 24.74120727 | 4.948241454 |
| PA0307 | PA0307 | 7 | 65 | 231.9488182 | 3.568443356 |
| PA0316 | serA | 13 | 21 | 85.04789999 | 4.0499 |
| PA0402 | pyrB | 9 | 1 | 12.37060363 | 12.37060363 |
| PA0407 | gshB | 10 | 7 | 26.28753272 | 3.755361818 |
| PA0466 | PA0466 | 1 | 0.1 | 27.83385818 | 278.3385818 |
| PA0526 | PA0526 | 1 | 38 | 160.8178473 | 4.232048612 |
| PA0553 | PA0553 | 2 | 0.1 | 12.37060363 | 123.7060363 |
| PA0712 | PA0712 | 4 | 7 | 24.74120727 | 3.534458181 |
| PA0721 | PA0721 | 4 | 0.1 | 12.37060363 | 123.7060363 |
| PA0766 | mucD | 11 | 101 | 400.4982927 | 3.965329631 |
| PA0909 | PA0909 | 5 | 34 | 108.2427818 | 3.18361123 |
| PA0928 | gacS | 18 | 3 | 9.277952726 | 3.092650909 |
| PA0945 | purM | 13 | 32 | 123.7060363 | 3.865813636 |
| PA0951a | PA0951a | 1 | 6 | 57.21404181 | 9.535673635 |
| PA0960 | PA0960 | 0 | 10 | 34.01916 | 3.401916 |
| PA0973 | oprL | 11 | 15 | 46.38976363 | 3.092650909 |
| PA0974 | PA0974 | 16 | 70 | 228.8561672 | 3.269373818 |
| PA1013 | purC | 3 | 2 | 15.46325454 | 7.731627272 |
| PA1034 | PA1034 | 2 | 6 | 40.20446181 | 6.700743636 |
| PA1101 | fliF | 16 | 421 | 1456.638578 | 3.459949116 |
| PA1103 | PA1103 | 2 | 0.1 | 35.56548545 | 355.6548545 |
| PA1121 | yfiR | 7 | 21 | 83.50157454 | 3.976265454 |
| PA1285 | PA1285 | 6 | 12 | 43.29711272 | 3.608092727 |
| PA1394 | PA1394 | 0 | 11 | 37.1118109 | 3.373800991 |
| PA1443 | fliM | 7 | 80 | 366.4791327 | 4.580989159 |
| PA1547 | PA1547 | 5 | 0.1 | 10.82427818 | 108.2427818 |
| PA1548 | PA1548 | 3 | 0.1 | 30.92650909 | 309.2650909 |
| PA1549 | PA1549 | 19 | 6 | 20.10223091 | 3.350371818 |
| PA1550 | PA1550 | 6 | 3 | 27.83385818 | 9.277952726 |
| PA1554 | ccoN1 | 25 | 211 | 638.6324127 | 3.026693899 |
| PA1615 | PA1615 | 5 | 11 | 35.56548545 | 3.23322595 |
| PA1629 | PA1629 | 5 | 21 | 80.40892363 | 3.828996363 |
| PA1632 | kdpF | 3 | 2 | 10.82427818 | 5.41213909 |
| PA1696 | pscO | 3 | 5 | 18.55590545 | 3.71118109 |
| PA1715 | pscB | 2 | 2 | 6.185301817 | 3.092650909 |
| PA1766 | PA1766 | 13 | 8 | 54.1213909 | 6.765173863 |
| PA1796.2 | PA1796.2 | 2 | 0.1 | 13.91692909 | 139.1692909 |
| PA1879 | PA1879 | 2 | 2 | 10.82427818 | 5.41213909 |
| PA2146 | PA2146 | 1 | 0.1 | 24.74120727 | 247.4120727 |
| PA2173 | PA2173 | 0 | 0.1 | 34.01916 | 340.1916 |
| PA2193 | hcnA | 2 | 1 | 12.37060363 | 12.37060363 |
| PA2297 | PA2297 | 3 | 17 | 58.76036727 | 3.456492192 |
| PA2330 | PA2330 | 3 | 2 | 21.64855636 | 10.82427818 |
| PA2425 | pvdG | 13 | 8 | 24.74120727 | 3.092650909 |
| PA2541 | PA2541 | 5 | 9 | 46.38976363 | 5.154418181 |
| PA2816 | PA2816 | 1 | 31 | 114.4280836 | 3.691228504 |
| PA2852 | PA2852 | 9 | 6 | 20.10223091 | 3.350371818 |
| PA2898 | PA2898 | 1 | 4 | 17.00958 | 4.252395 |
| PA2947 | PA2947 | 2 | 18 | 69.58464545 | 3.865813636 |
| PA2964 | pabC | 5 | 5 | 27.83385818 | 5.566771636 |
| PA2992 | PA2992 | 2 | 25 | 119.06706 | 4.762682399 |
| PA3094.2 | PA3094.2 | 3 | 2 | 12.37060363 | 6.185301817 |
| PA3099 | xcpV | 1 | 8 | 29.38018363 | 3.672522954 |
| PA3111 | folC | 15 | 5 | 20.10223091 | 4.020446181 |
| PA3173 | PA3173 | 11 | 11 | 44.84343818 | 4.076676198 |
| PA3202 | PA3202 | 3 | 10 | 34.01916 | 3.401916 |
| PA3299 | fadD1 | 19 | 22 | 98.96482908 | 4.498401322 |
| PA3382 | phnE | 4 | 32 | 128.3450127 | 4.010781647 |
| PA3509 | PA3509 | 6 | 0.1 | 27.83385818 | 278.3385818 |
| PA3574a | PA3574a | 3 | 11 | 58.76036727 | 5.34185157 |
| PA3738 | xerD | 9 | 9 | 34.01916 | 3.779906666 |
| PA3815 | iscR | 7 | 13 | 51.02873999 | 3.925287692 |
| PA4232 | ssb | 6 | 0.1 | 12.37060363 | 123.7060363 |
| PA4275 | nusG | 9 | 8 | 61.85301817 | 7.731627272 |
| PA4351 | PA4351 | 4 | 9 | 41.75078727 | 4.638976363 |
| PA4357 | PA4357 | 1 | 21 | 80.40892363 | 3.828996363 |
| PA4431 | PA4431 | 8 | 2 | 17.00958 | 8.504789999 |
| PA4451 | PA4451 | 2 | 5 | 29.38018363 | 5.876036727 |
| PA4726 | cbrB | 14 | 181 | 889.1371363 | 4.912359869 |
| PA4726.1 | PA4726.1 | 4 | 1 | 12.37060363 | 12.37060363 |
| PA4752 | ftsJ | 9 | 9 | 32.47283454 | 3.608092727 |
| PA4782 | PA4782 | 2 | 39 | 119.06706 | 3.053001538 |
| PA4853 | fis | 4 | 1 | 40.20446181 | 40.20446181 |
| PA4890 | desT | 2 | 0.1 | 12.37060363 | 123.7060363 |
| PA4894 | PA4894 | 3 | 8 | 34.01916 | 4.252395 |
| PA5000 | wapR | 21 | 6 | 24.74120727 | 4.123534545 |
| PA5015 | aceE | 38 | 58 | 270.6069545 | 4.665637147 |
| PA5016 | aceF | 7 | 3 | 18.55590545 | 6.185301817 |
| PA5113 | PA5113 | 15 | 198 | 632.4471108 | 3.194177327 |
| PA5114 | PA5114 | 37 | 218 | 3002.964032 | 13.77506437 |
| PA5117 | typA | 13 | 13 | 66.49199454 | 5.114768811 |
| PA5148 | PA5148 | 5 | 33 | 149.9935691 | 4.545259669 |
| PA5162 | rmlD | 11 | 6 | 40.20446181 | 6.700743636 |
| PA5198 | PA5198 | 9 | 4 | 12.37060363 | 3.092650909 |
| PA5260 | hemC | 8 | 0.1 | 12.37060363 | 123.7060363 |
| PA5285 | PA5285 | 1 | 8 | 57.21404181 | 7.151755226 |
| PA5366 | pstB | 11 | 1 | 6.185301817 | 6.185301817 |
|  | | | | | |
| *Genes with higher insertion frequency + B.t. WT* | | | | | |
| PA0006 | PA0006 | 13 | 156 | 34.01916 | 0.218071538 |
| PA0125 | PA0125 | 2 | 40 | 12.37060363 | 0.309265091 |
| PA0871 | phhB | 4 | 225 | 41.75078727 | 0.185559055 |
| PA1045 | PA1045 | 17 | 6 | 1.546325454 | 0.257720909 |
| PA1112a | PA1112a | 1 | 55 | 12.37060363 | 0.224920066 |
| PA1117 | PA1117 | 1 | 47 | 12.37060363 | 0.263204333 |
| PA1149 | PA1149 | 3 | 86 | 20.10223091 | 0.233746871 |
| PA1446 | fliP | 3 | 161 | 46.38976363 | 0.288135178 |
| PA1503 | PA1503 | 4 | 78 | 23.19488182 | 0.29737028 |
| PA1571 | PA1571 | 2 | 48 | 7.731627272 | 0.161075568 |
| PA1664 | PA1664 | 2 | 17 | 0.1 | 0.005882353 |
| PA1685 | masA | 10 | 30 | 9.277952726 | 0.309265091 |
| PA1742 | PA1742 | 12 | 129 | 32.47283454 | 0.2517274 |
| PA1743 | PA1743 | 3 | 42 | 6.185301817 | 0.147269091 |
| PA1802 | clpX | 20 | 19 | 6.185301817 | 0.325542201 |
| PA1842 | PA1842 | 2 | 72 | 23.19488182 | 0.322151136 |
| PA1995 | PA1995 | 3 | 33 | 10.82427818 | 0.32800843 |
| PA2003 | bdhA | 3 | 112 | 35.56548545 | 0.317548977 |
| PA2007 | maiA | 8 | 455 | 106.6964564 | 0.234497706 |
| PA2008 | fahA | 11 | 174 | 18.55590545 | 0.106643135 |
| PA2143 | PA2143 | 2 | 88 | 10.82427818 | 0.123003161 |
| PA2183 | PA2183 | 3 | 58 | 18.55590545 | 0.319929404 |
| PA2187 | PA2187 | 6 | 116 | 24.74120727 | 0.21328627 |
| PA2357 | msuE | 7 | 112 | 37.1118109 | 0.331355455 |
| PA2492 | mexT | 8 | 17782 | 4741.033843 | 0.266619831 |
| PA2614 | lolA | 5 | 63 | 20.10223091 | 0.31908303 |
| PA2967 | fabG | 8 | 11 | 1.546325454 | 0.140575041 |
| PA3139.1 | PA3139.1 | 3 | 17 | 0.1 | 0.005882353 |
| PA3165 | hisC2 | 15 | 32 | 0.1 | 0.003125 |
| PA3368.1 | PA3368.1 | 3 | 35 | 6.185301817 | 0.176722909 |
| PA3520 | PA3520 | 4 | 56 | 13.91692909 | 0.248516591 |
| PA3557 | arnE | 1 | 18 | 3.092650909 | 0.171813939 |
| PA3721 | nalC | 8 | 6 | 1.546325454 | 0.257720909 |
| PA3808 | PA3808 | 2 | 46 | 12.37060363 | 0.268926166 |
| PA3813 | iscU | 3 | 39 | 1.546325454 | 0.039649371 |
| PA3821 | secD | 16 | 10 | 0.1 | 0.01 |
| PA3822 | PA3822 | 3 | 12 | 0.1 | 0.008333333 |
| PA4092 | hpaC | 1 | 38 | 6.185301817 | 0.1627711 |
| PA4236 | katA | 23 | 2815 | 896.8687635 | 0.318603468 |
| PA4561 | ribF | 7 | 27 | 4.638976363 | 0.171813939 |
| PA4662 | murI | 15 | 30 | 3.092650909 | 0.103088364 |
| PA4758 | carA | 17 | 5 | 1.546325454 | 0.309265091 |
| PA5062 | PA5062 | 0 | 21 | 3.092650909 | 0.147269091 |
| PA5134 | PA5134 | 11 | 319 | 94.32585272 | 0.295692328 |
