# Supplementary material for: Discovery of coordinately regulated pathways that provide innate protection against interbacterial antagonism
Source: eLife. 2022 Feb 17;11:e74658. doi: 10.7554/eLife.74658 (PMC8926400; doi:10.7554/eLife.74658)
Supplement: Figure 2—source data 2. [file elife-74658-fig2-data2.docx]

Figure 2­Source Data 2. Transposon sequencing-based analysis of *P. aeruginosa* fitness determinants during antagonism by *B. thai* compared to *B. thai* ∆*colA.*

|  |  |  | Normalized insertion counts | |  |
| --- | --- | --- | --- | --- | --- |
| Locus ID | Gene name | TA sites | + *B.t.* WT | + *B.t*. ∆*colA* | Fold change (∆*colA*/WT) |
| *Genes with higher insertion frequency + B.t. ∆colA* | | | | | |
| PA0005 | lptA | 11 | 2 | 7.928575216 | 3.964287608 |
| PA0037 | trpI | 8 | 237 | 767.4860809 | 3.238337894 |
| PA0180 | cttP | 5 | 3 | 17.44286548 | 5.814288492 |
| PA0197 | tonB2 | 2 | 5 | 25.37144069 | 5.074288138 |
| PA0407 | gshB | 10 | 7 | 25.37144069 | 3.624491527 |
| PA0466 | PA0466 | 1 | 0 | 11.1000053 | N.D. |
| PA0526 | PA0526 | 1 | 38 | 185.5286601 | 4.88233316 |
| PA0712 | PA0712 | 4 | 7 | 33.30001591 | 4.75714513 |
| PA0753 | PA0753 | 5 | 15 | 63.42860173 | 4.228573449 |
| PA0928 | gacS | 18 | 3 | 9.51429026 | 3.171430087 |
| PA0947 | PA0947 | 7 | 7 | 22.20001061 | 3.171430087 |
| PA0960 | PA0960 | 0 | 10 | 44.40002121 | 4.440002121 |
| PA0973 | oprL | 11 | 15 | 57.08574156 | 3.805716104 |
| PA1034 | PA1034 | 2 | 6 | 49.15716634 | 8.192861057 |
| PA1045 | PA1045 | 17 | 6 | 30.12858582 | 5.02143097 |
| PA1121 | yfiR | 7 | 21 | 77.70003712 | 3.700001768 |
| PA1233 | PA1233 | 5 | 16 | 49.15716634 | 3.072322896 |
| PA1394 | PA1394 | 0 | 11 | 33.30001591 | 3.027274174 |
| PA1548 | PA1548 | 3 | 0 | 14.27143539 | N.D. |
| PA1550 | PA1550 | 6 | 3 | 9.51429026 | 3.171430087 |
| PA1552.1 | ccoQ1 | 2 | 0 | 26.95715574 | N.D. |
| PA1564 | PA1564 | 2 | 0 | 12.68572035 | N.D. |
| PA1645 | PA1645 | 5 | 9 | 36.471446 | 4.052382888 |
| PA1696 | pscO | 3 | 5 | 42.81430617 | 8.562861234 |
| PA1747 | PA1747 | 1 | 0 | 15.85715043 | N.D. |
| PA1796.2 | PA1796.2 | 2 | 0 | 12.68572035 | N.D. |
| PA1816 | dnaQ | 8 | 0 | 15.85715043 | N.D. |
| PA1879 | PA1879 | 2 | 2 | 19.02858052 | 9.51429026 |
| PA2176 | PA2176 | 4 | 17 | 52.32859643 | 3.078152731 |
| PA2193 | hcnA | 2 | 1 | 20.61429556 | 20.61429556 |
| PA2321 | PA2321 | 3 | 1 | 26.95715574 | 26.95715574 |
| PA2330 | PA2330 | 3 | 2 | 11.1000053 | 5.550002651 |
| PA2425 | pvdG | 13 | 8 | 39.64287608 | 4.95535951 |
| PA2541 | PA2541 | 5 | 9 | 30.12858582 | 3.347620647 |
| PA2554 | PA2554 | 3 | 11 | 47.5714513 | 4.324677391 |
| PA2724 | PA2724 | 4 | 41 | 161.7429344 | 3.94494962 |
| PA2738 | himA | 3 | 4 | 12.68572035 | 3.171430087 |
| PA2743 | infC | 12 | 2 | 6.342860173 | 3.171430087 |
| PA2805 | PA2805 | 0 | 6 | 20.61429556 | 3.435715927 |
| PA2855 | PA2855 | 3 | 15 | 45.98573625 | 3.06571575 |
| PA2895 | PA2895 | 1 | 9 | 28.54287078 | 3.171430087 |
| PA2898 | PA2898 | 1 | 4 | 15.85715043 | 3.964287608 |
| PA2914 | PA2914 | 5 | 33 | 115.7571982 | 3.507793884 |
| PA2947 | PA2947 | 2 | 18 | 63.42860173 | 3.523811207 |
| PA2964 | pabC | 5 | 5 | 19.02858052 | 3.805716104 |
| PA2991 | sth | 18 | 0 | 14.27143539 | N.D. |
| PA2992 | PA2992 | 2 | 25 | 98.31433268 | 3.932573307 |
| PA3051 | PA3051 | 2 | 5 | 17.44286548 | 3.488573095 |
| PA3067 | PA3067 | 1 | 0 | 25.37144069 | N.D. |
| PA3094.2 | PA3094.2 | 3 | 2 | 23.78572565 | 11.89286282 |
| PA3099 | xcpV | 1 | 8 | 34.88573095 | 4.360716369 |
| PA3111 | folC | 15 | 5 | 42.81430617 | 8.562861234 |
| PA3133.4 | PA3133.4 | 3 | 0 | 14.27143539 | N.D. |
| PA3145 | wbpL | 34 | 1 | 9.51429026 | 9.51429026 |
| PA3205 | PA3205 | 1 | 5 | 20.61429556 | 4.122859113 |
| PA3382 | phnE | 4 | 32 | 115.7571982 | 3.617412442 |
| PA3385 | amrZ | 3 | 20 | 61.84288669 | 3.092144334 |
| PA3574a | PA3574a | 3 | 11 | 53.91431147 | 4.901301043 |
| PA3634 | PA3634 | 2 | 4 | 14.27143539 | 3.567858847 |
| PA3640 | dnaE | 46 | 2 | 15.85715043 | 7.928575216 |
| PA3649 | PA3649 | 20 | 22 | 74.52860703 | 3.387663956 |
| PA3815 | iscR | 7 | 13 | 49.15716634 | 3.781320488 |
| PA3879 | narL | 2 | 31 | 123.6857734 | 3.989863657 |
| PA4020 | mpl | 15 | 1 | 3.171430087 | 3.171430087 |
| PA4275 | nusG | 9 | 8 | 71.35717695 | 8.919647118 |
| PA4277 | tufB | 23 | 2 | 6.342860173 | 3.171430087 |
| PA4319 | PA4319 | 13 | 95 | 307.6287184 | 3.238197036 |
| PA4320 | PA4320 | 19 | 70 | 274.3287025 | 3.918981464 |
| PA4322 | PA4322 | 6 | 22 | 115.7571982 | 5.261690825 |
| PA4323 | PA4323 | 15 | 27 | 107.8286229 | 3.993652702 |
| PA4351 | PA4351 | 4 | 9 | 47.5714513 | 5.285716811 |
| PA4413 | ftsW | 12 | 9 | 50.74288138 | 5.638097932 |
| PA4422 | PA4422 | 6 | 0 | 14.27143539 | N.D. |
| PA4431 | PA4431 | 8 | 2 | 6.342860173 | 3.171430087 |
| PA4452 | PA4452 | 2 | 11 | 49.15716634 | 4.468833304 |
| PA4459 | PA4459 | 13 | 9 | 31.71430087 | 3.523811207 |
| PA4610 | PA4610 | 1 | 17 | 53.91431147 | 3.171430087 |
| PA4671 | PA4671 | 7 | 2 | 11.1000053 | 5.550002651 |
| PA4672 | PA4672 | 5 | 3 | 12.68572035 | 4.228573449 |
| PA4726.1 | PA4726.1 | 4 | 1 | 17.44286548 | 17.44286548 |
| PA4820 | PA4820 | 5 | 9 | 26.95715574 | 2.995239526 |
| PA4853 | fis | 4 | 1 | 19.02858052 | 19.02858052 |
| PA4890 | desT | 2 | 0 | 20.61429556 | N.D. |
| PA4894 | PA4894 | 3 | 8 | 52.32859643 | 6.541074553 |
| PA4933 | PA4933 | 10 | 7 | 22.20001061 | 3.171430087 |
| PA4952 | PA4952 | 5 | 4 | 26.95715574 | 6.739288934 |
| PA5000 | wapR | 21 | 6 | 25.37144069 | 4.228573449 |
| PA5016 | aceF | 7 | 3 | 22.20001061 | 7.400003535 |
| PA5049 | rpmE | 7 | 4 | 22.20001061 | 5.550002651 |
| PA5148 | PA5148 | 5 | 33 | 111.000053 | 3.363637971 |
| PA5162 | rmlD | 11 | 6 | 45.98573625 | 7.664289376 |
| PA5198 | PA5198 | 9 | 4 | 17.44286548 | 4.360716369 |
| PA5199 | amgS | 9 | 3 | 23.78572565 | 7.928575216 |
| PA5285 | PA5285 | 1 | 8 | 34.88573095 | 4.360716369 |
|  | | | | | |
| *Genes with higher insertion frequency + B.t.* WT | | | | | |
| PA0006 | PA0006 | 13 | 156 | 41.22859113 | 0.264285841 |
| PA0125 | PA0125 | 2 | 40 | 7.928575216 | 0.19821438 |
| PA0420 | bioA | 19 | 45 | 12.68572035 | 0.281904897 |
| PA0666 | PA0666 | 10 | 127 | 36.471446 | 0.28717674 |
| PA0715 | PA0715 | 61 | 14 | 3.171430087 | 0.22653072 |
| PA0763 | mucA | 9 | 52 | 15.85715043 | 0.304945201 |
| PA0905.3 | PA0905.3 | 4 | 10 | 1.585715043 | 0.158571504 |
| PA0913 | mgtE | 10 | 96 | 23.78572565 | 0.247767976 |
| PA1030.1 | PA1030.1 | 2 | 55 | 15.85715043 | 0.288311826 |
| PA1117 | PA1117 | 1 | 47 | 1.585715043 | 0.033738618 |
| PA1149 | PA1149 | 3 | 86 | 12.68572035 | 0.147508376 |
| PA1159 | PA1159 | 3 | 32 | 9.51429026 | 0.297321571 |
| PA1801 | clpP | 12 | 22 | 6.342860173 | 0.288311826 |
| PA1845 | PA1845 | 4 | 184 | 58.6714566 | 0.318866612 |
| PA1985 | pqqA | 1 | 12 | 0.1 | 0.008333333 |
| PA2142a | PA2142a | 2 | 50 | 9.51429026 | 0.190285805 |
| PA2187 | PA2187 | 6 | 116 | 36.471446 | 0.314409017 |
| PA2375 | PA2375 | 1 | 32 | 9.51429026 | 0.297321571 |
| PA2421 | PA2421 | 16 | 302 | 99.90004773 | 0.33079486 |
| PA2645 | nuoJ | 8 | 145 | 45.98573625 | 0.317143009 |
| PA2778 | PA2778 | 7 | 325 | 104.6571929 | 0.322022132 |
| PA2967 | fabG | 8 | 11 | 0.1 | 0.009090909 |
| PA2970 | rpmF | 5 | 30 | 7.928575216 | 0.264285841 |
| PA3014 | faoA | 19 | 17 | 0.1 | 0.005882353 |
| PA3139.1 | PA3139.1 | 3 | 17 | 4.75714513 | 0.279832066 |
| PA3151 | hisF2 | 27 | 19 | 3.171430087 | 0.166917373 |
| PA3165 | hisC2 | 15 | 32 | 9.51429026 | 0.297321571 |
| PA3482 | metG | 25 | 71 | 20.61429556 | 0.290342191 |
| PA3496 | PA3496 | 1 | 71 | 12.68572035 | 0.178672118 |
| PA3808 | PA3808 | 2 | 46 | 6.342860173 | 0.137888265 |
| PA3821 | secD | 16 | 10 | 3.171430087 | 0.317143009 |
| PA3844 | PA3844 | 8 | 898 | 247.3715468 | 0.275469428 |
| PA4092 | hpaC | 1 | 38 | 0 | 0 |
| PA4236 | katA | 23 | 2815 | 829.3289676 | 0.294610646 |
| PA4479 | mreD | 6 | 56 | 17.44286548 | 0.311479741 |
| PA4581.1 | PA4581.1 | 2 | 21 | 6.342860173 | 0.302040961 |
| PA4663 | moeB | 9 | 28 | 3.171430087 | 0.11326536 |
| PA5114 | PA5114 | 37 | 218 | 55.50002651 | 0.254587278 |
| PA5134 | PA5134 | 11 | 319 | 71.35717695 | 0.22369021 |
| PA5223 | ubiH | 14 | 36 | 7.928575216 | 0.2202382 |
| PA5241 | ppx | 15 | 10 | 3.171430087 | 0.317143009 |
