# Supplementary figures and images for: Discovery of coordinately regulated pathways that provide innate protection against interbacterial antagonism
Source: eLife. 2022 Feb 17;11:e74658. doi: 10.7554/eLife.74658 (PMC8926400; doi:10.7554/eLife.74658)

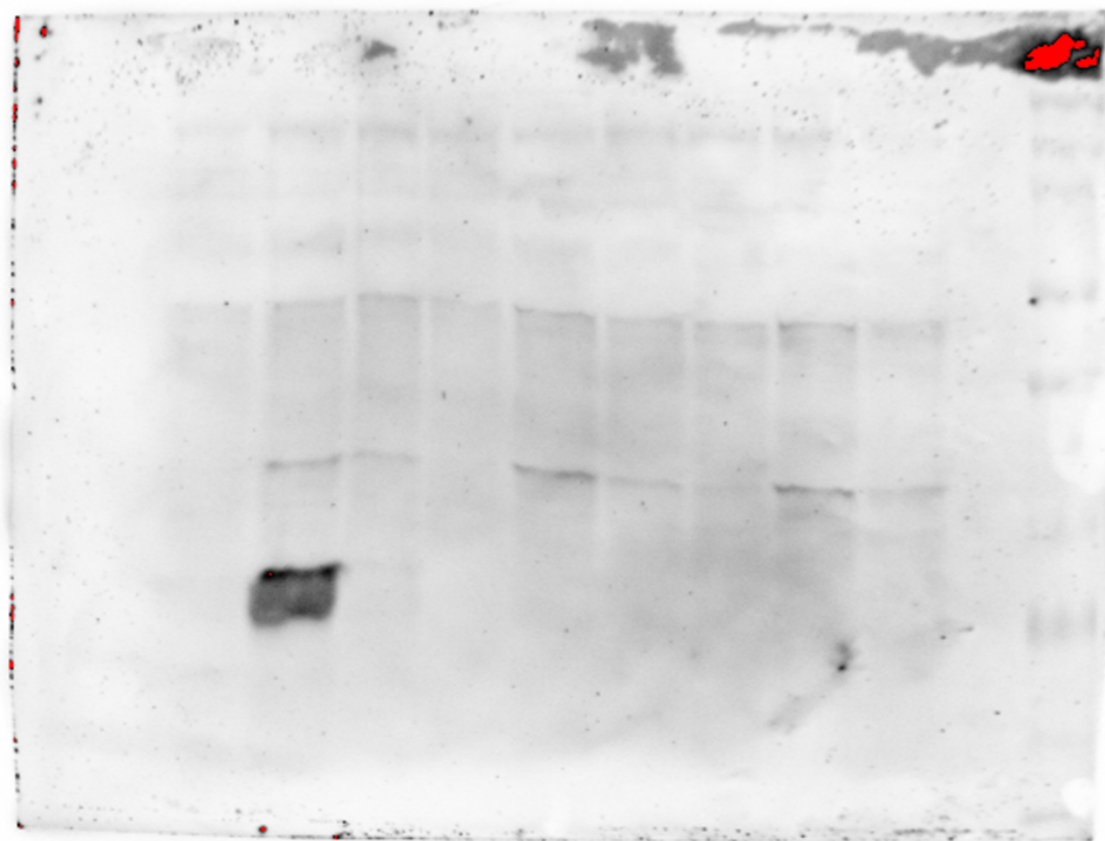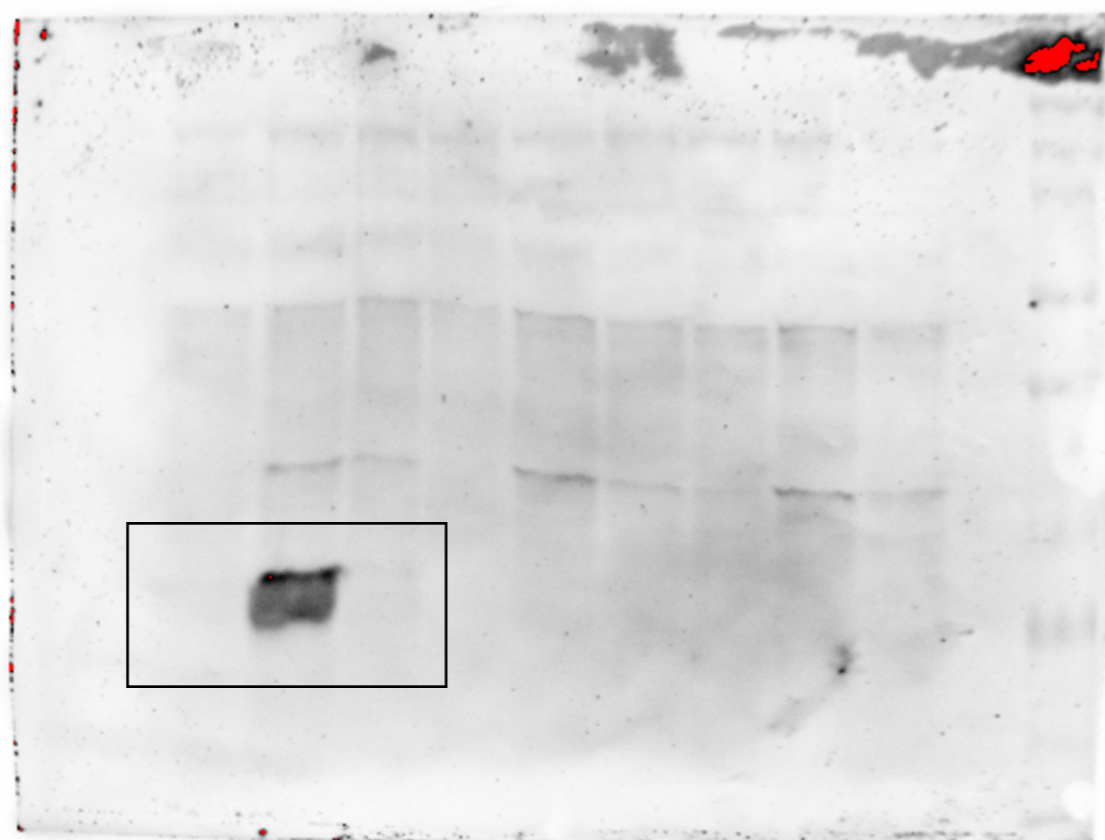

Supplement: Figure 2—figure supplement 2—source data 1. [file elife-74658-fig2-figsupp2-data1.pdf]

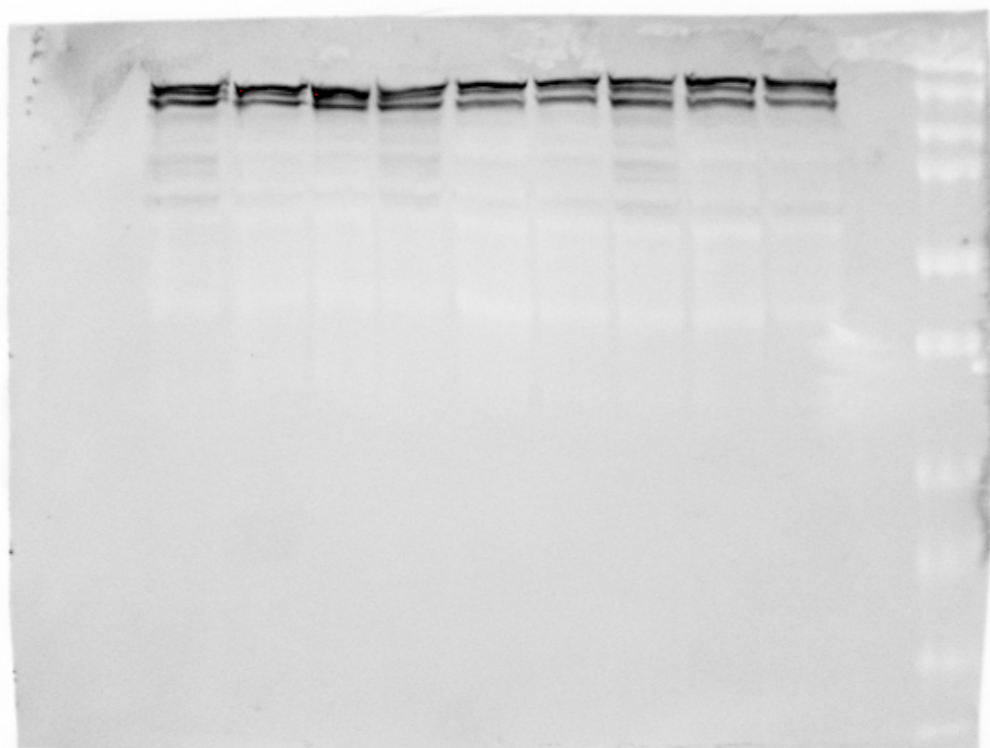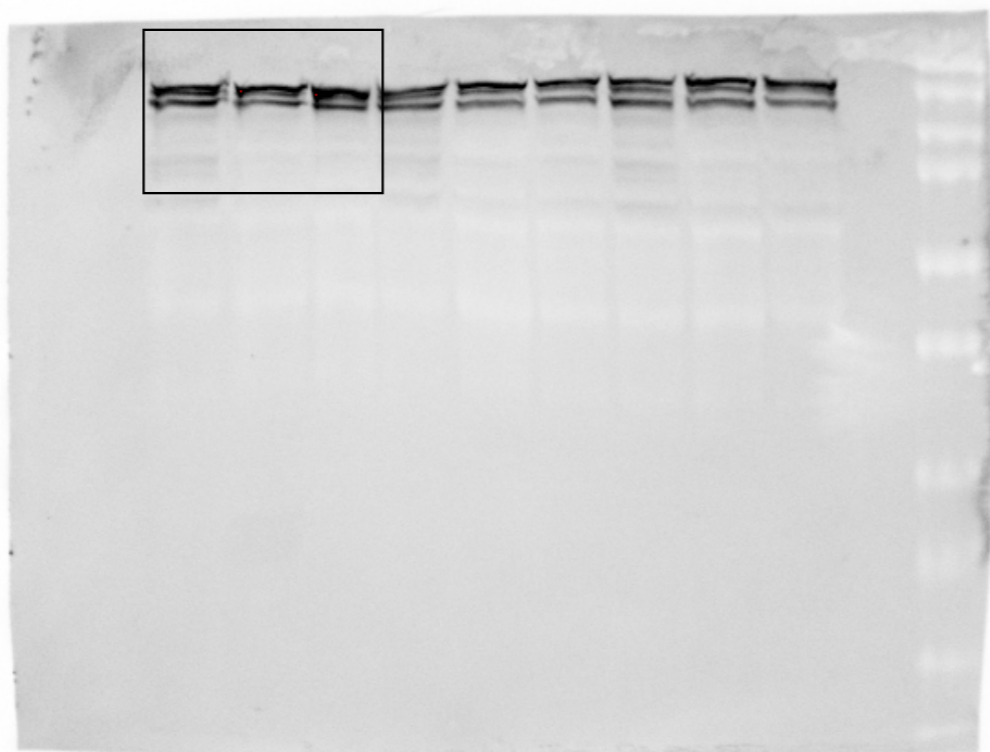

Supplement: Figure 2—figure supplement 2—source data 2. [file elife-74658-fig2-figsupp2-data2.pdf]

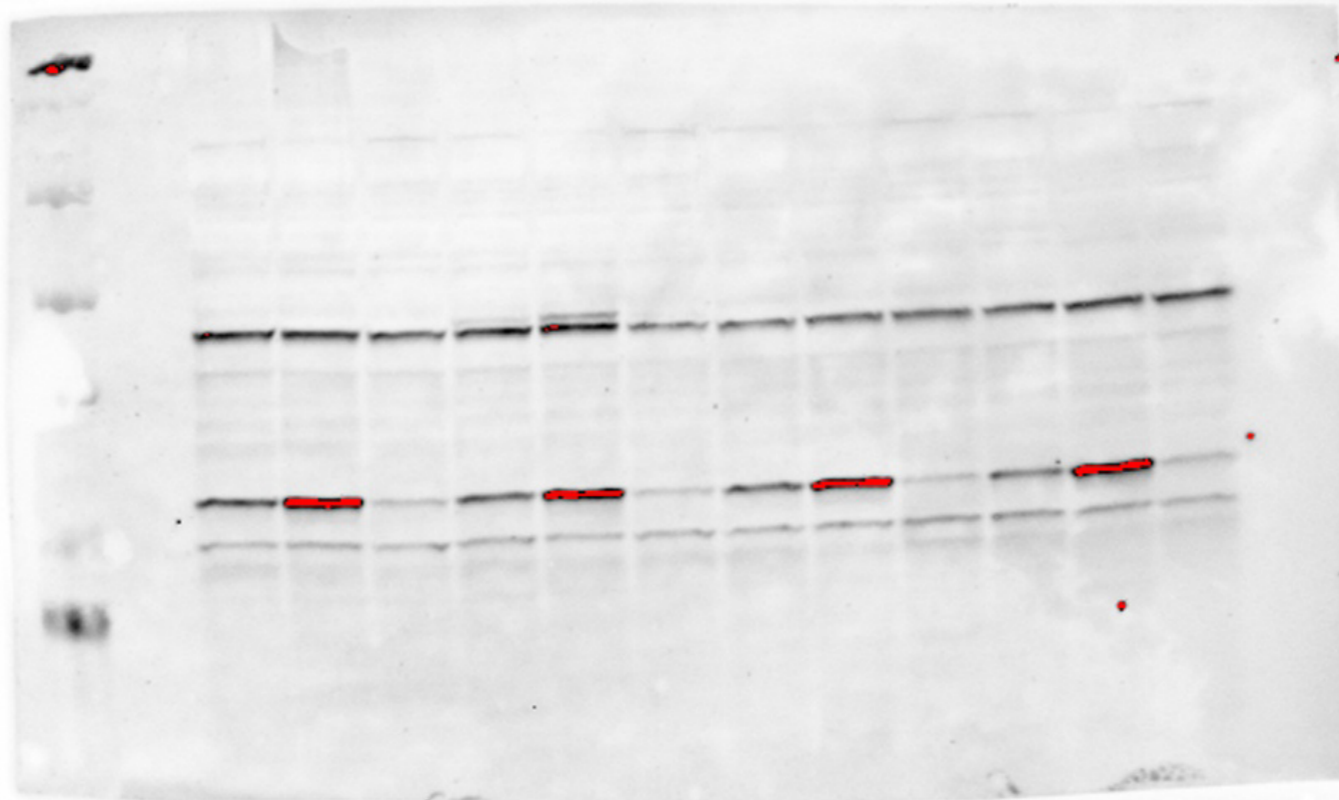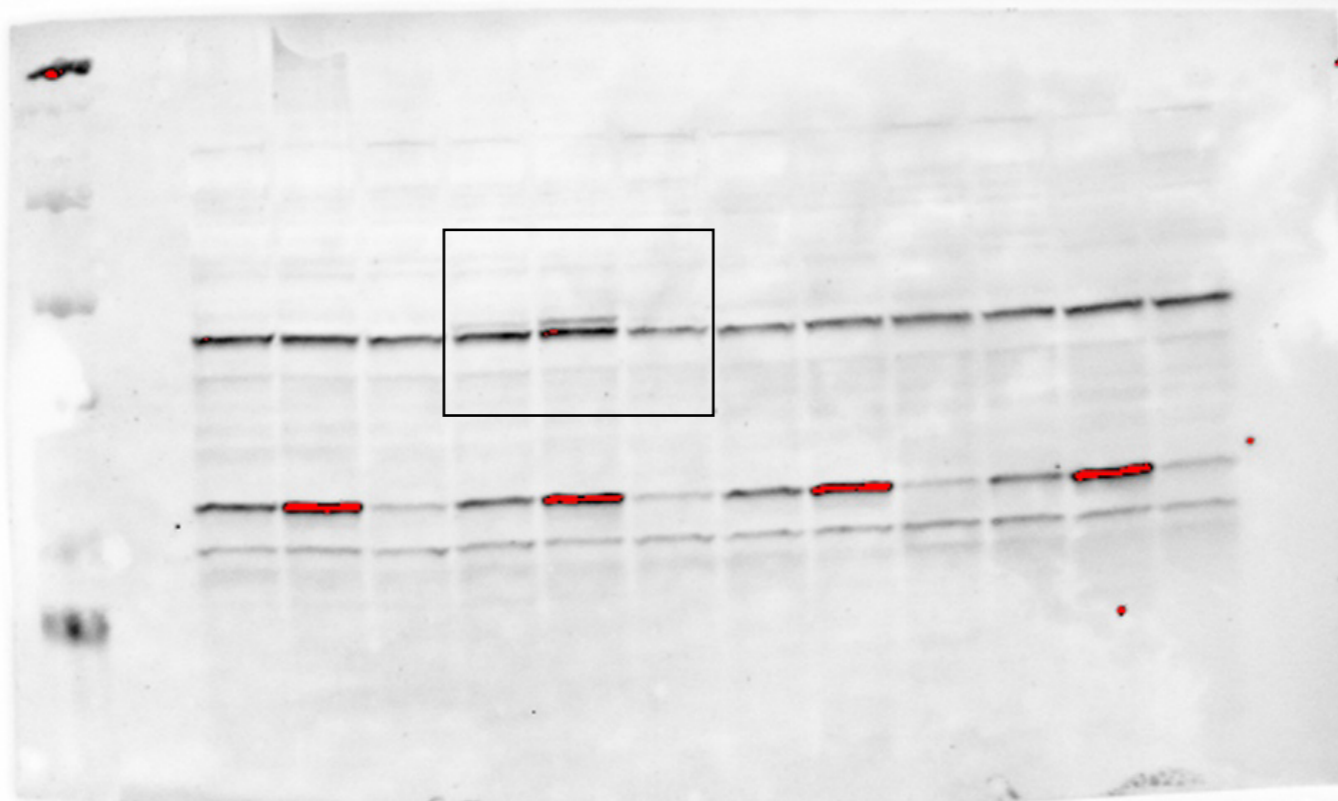

Supplement: Figure 2—figure supplement 2—source data 3. [file elife-74658-fig2-figsupp2-data3.pdf]

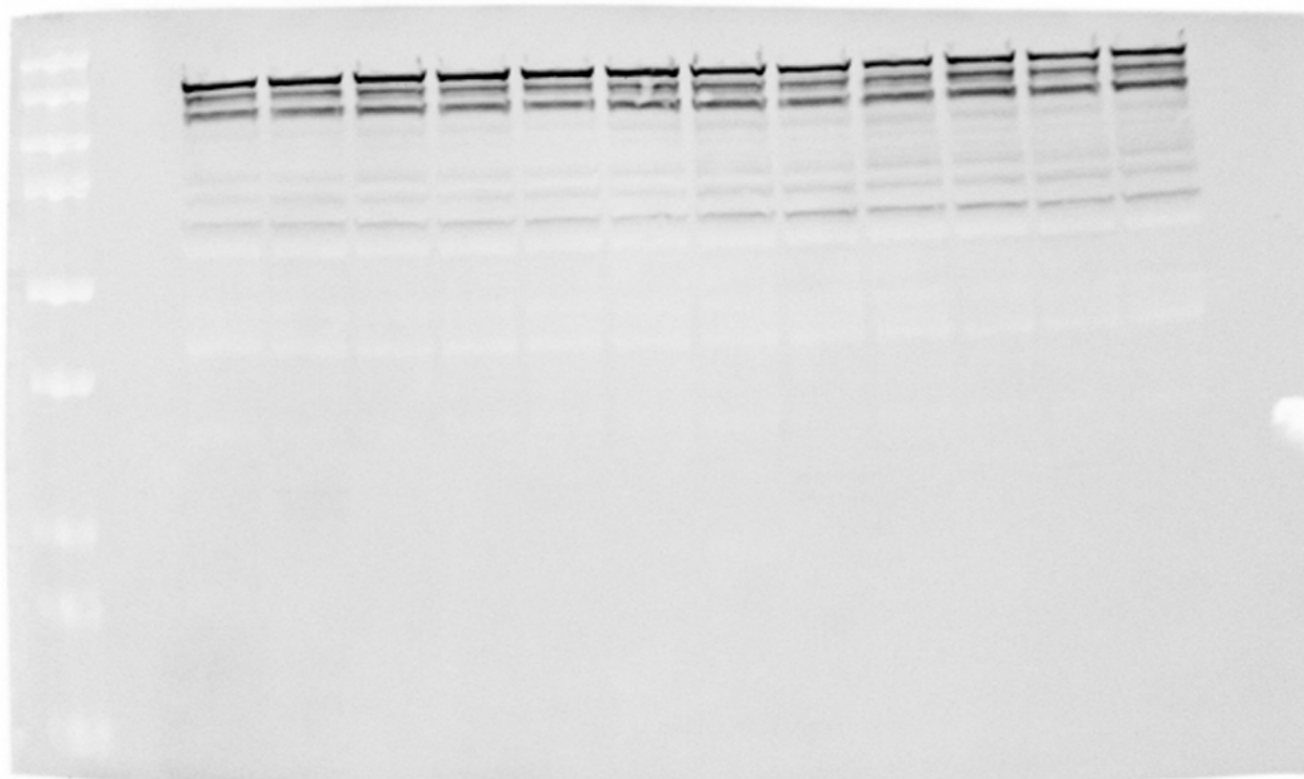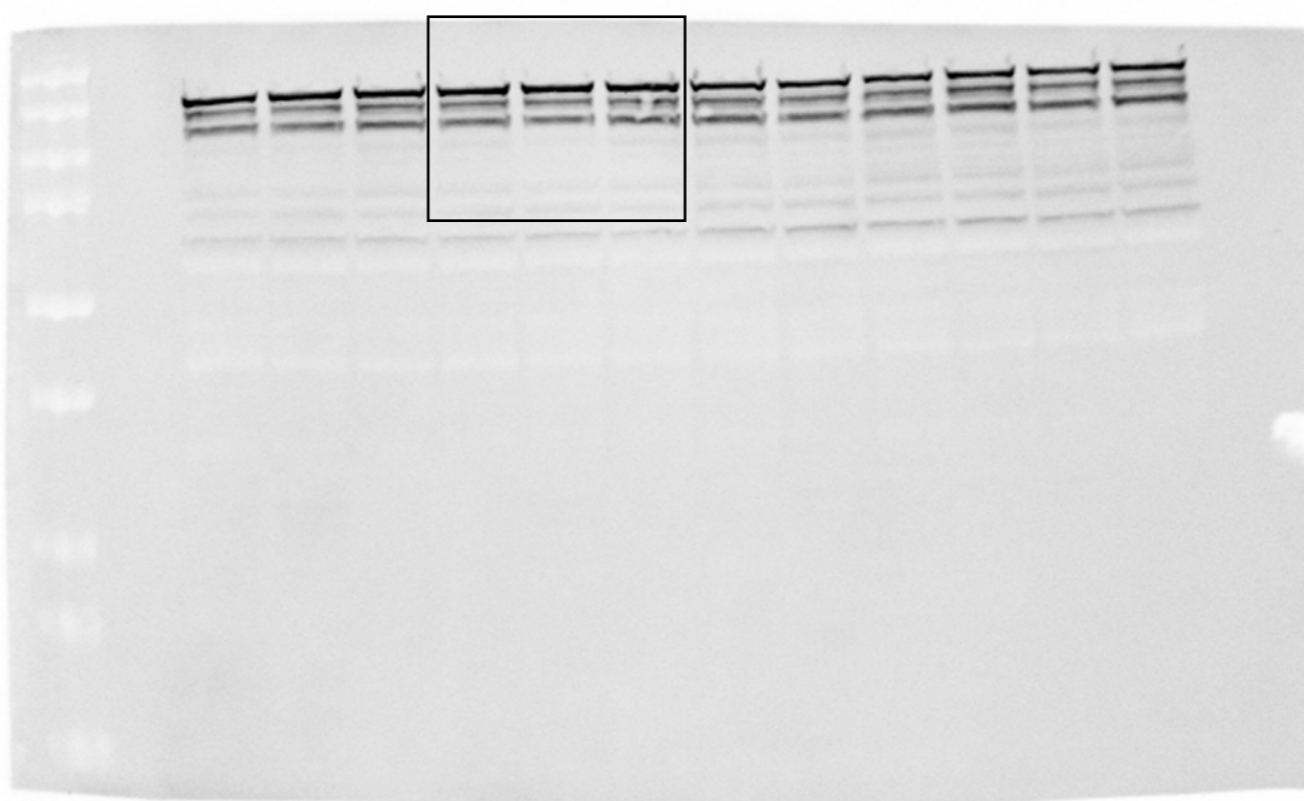

Supplement: Figure 2—figure supplement 2—source data 4. [file elife-74658-fig2-figsupp2-data4.pdf]

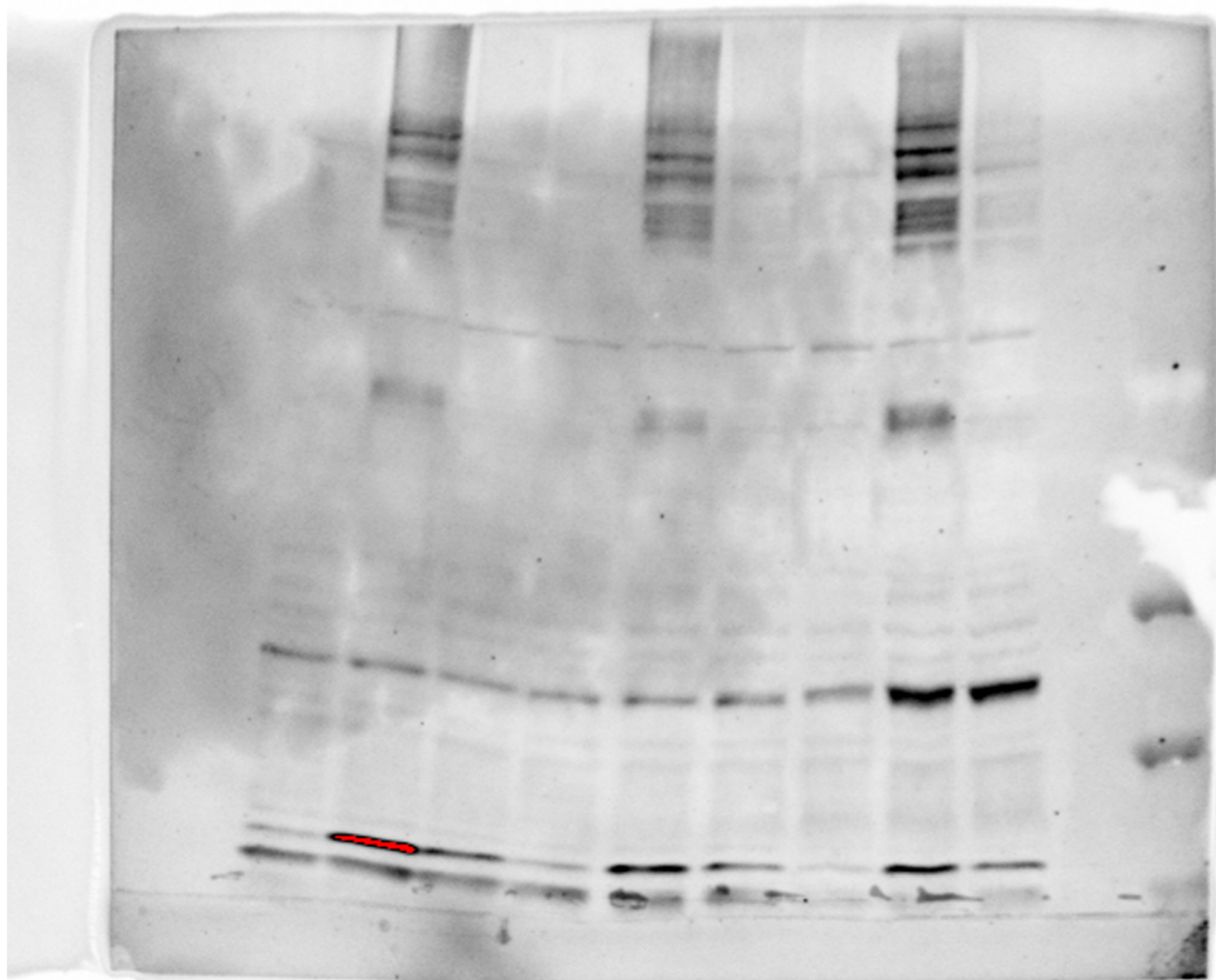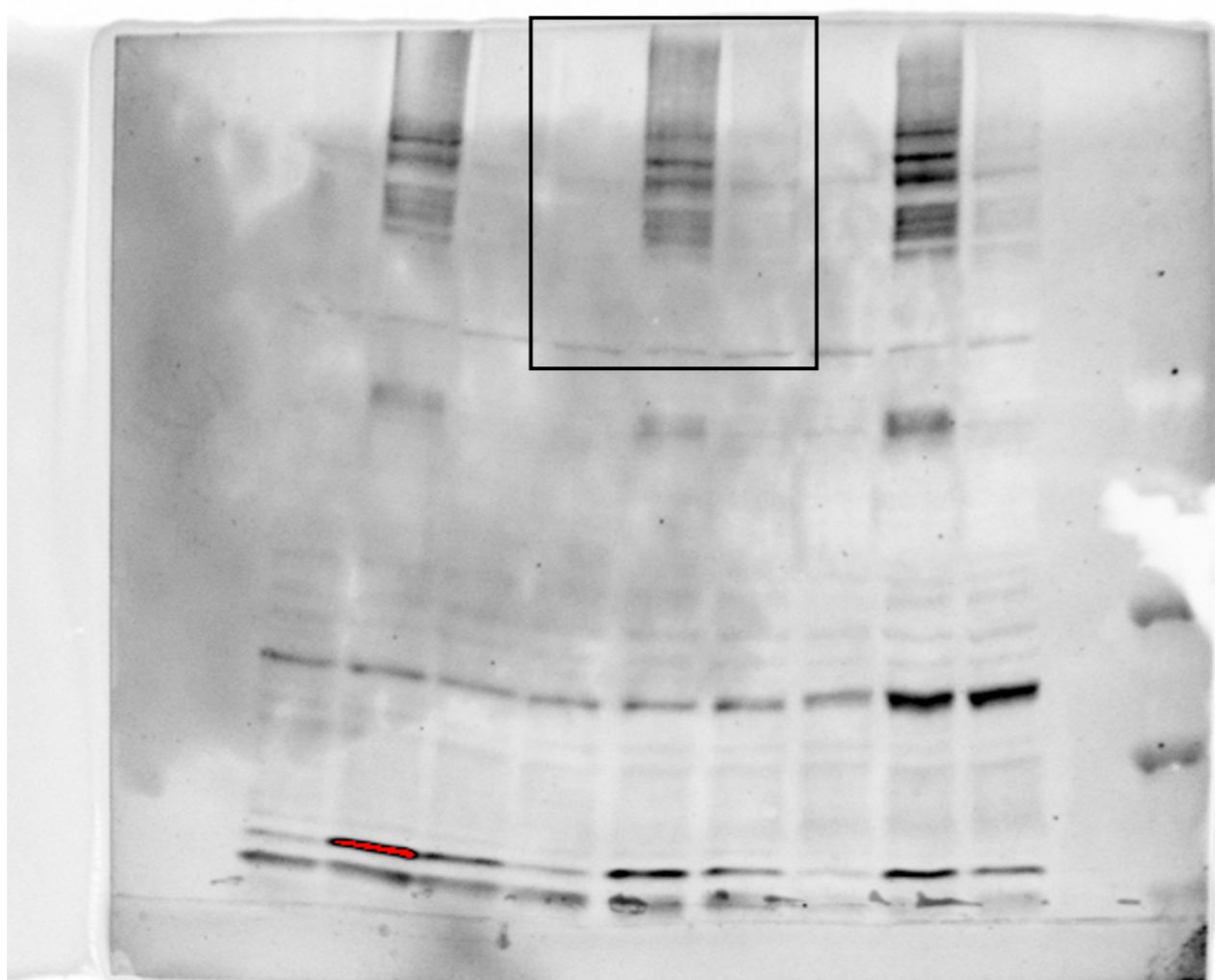

Supplement: Figure 2—figure supplement 2—source data 5. [file elife-74658-fig2-figsupp2-data5.pdf]

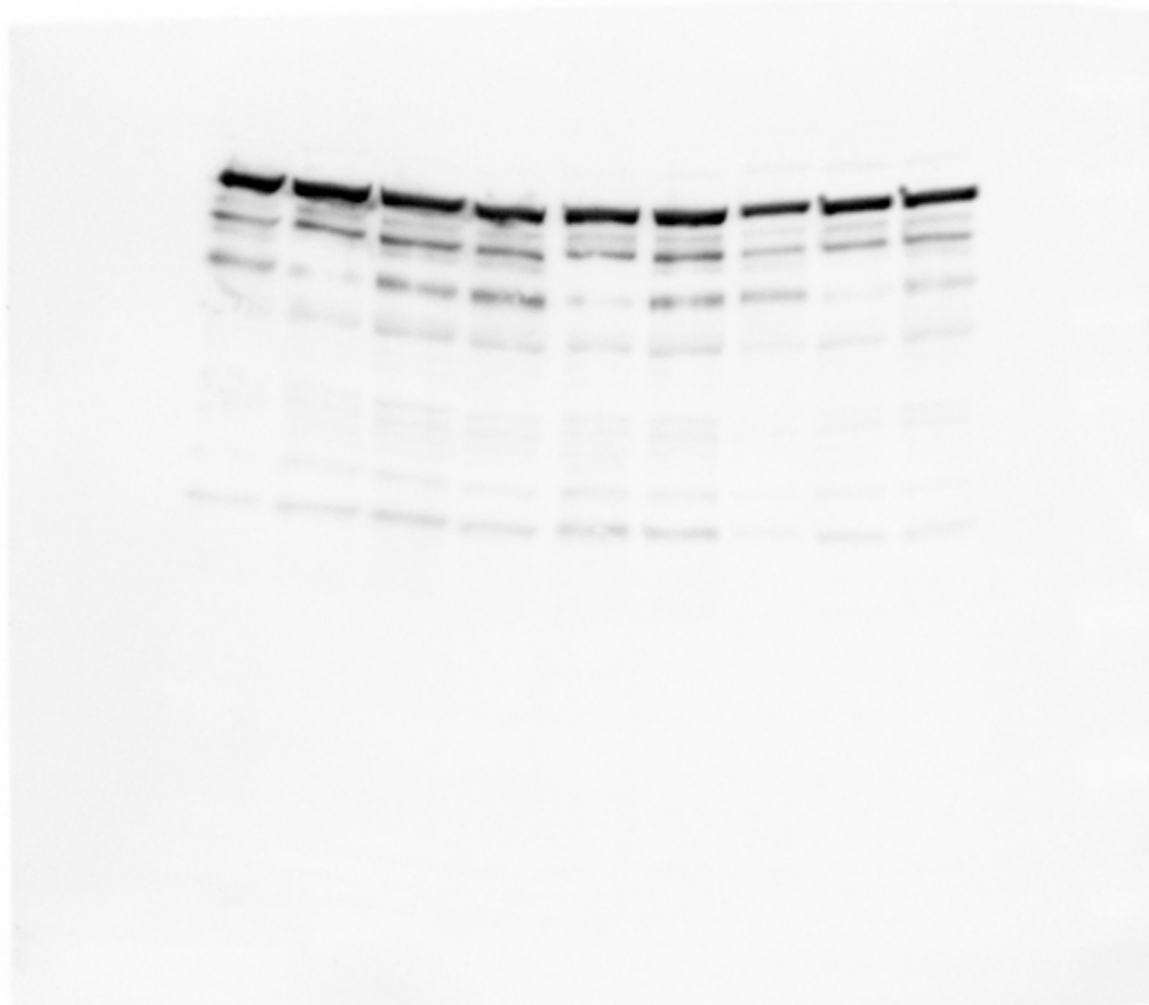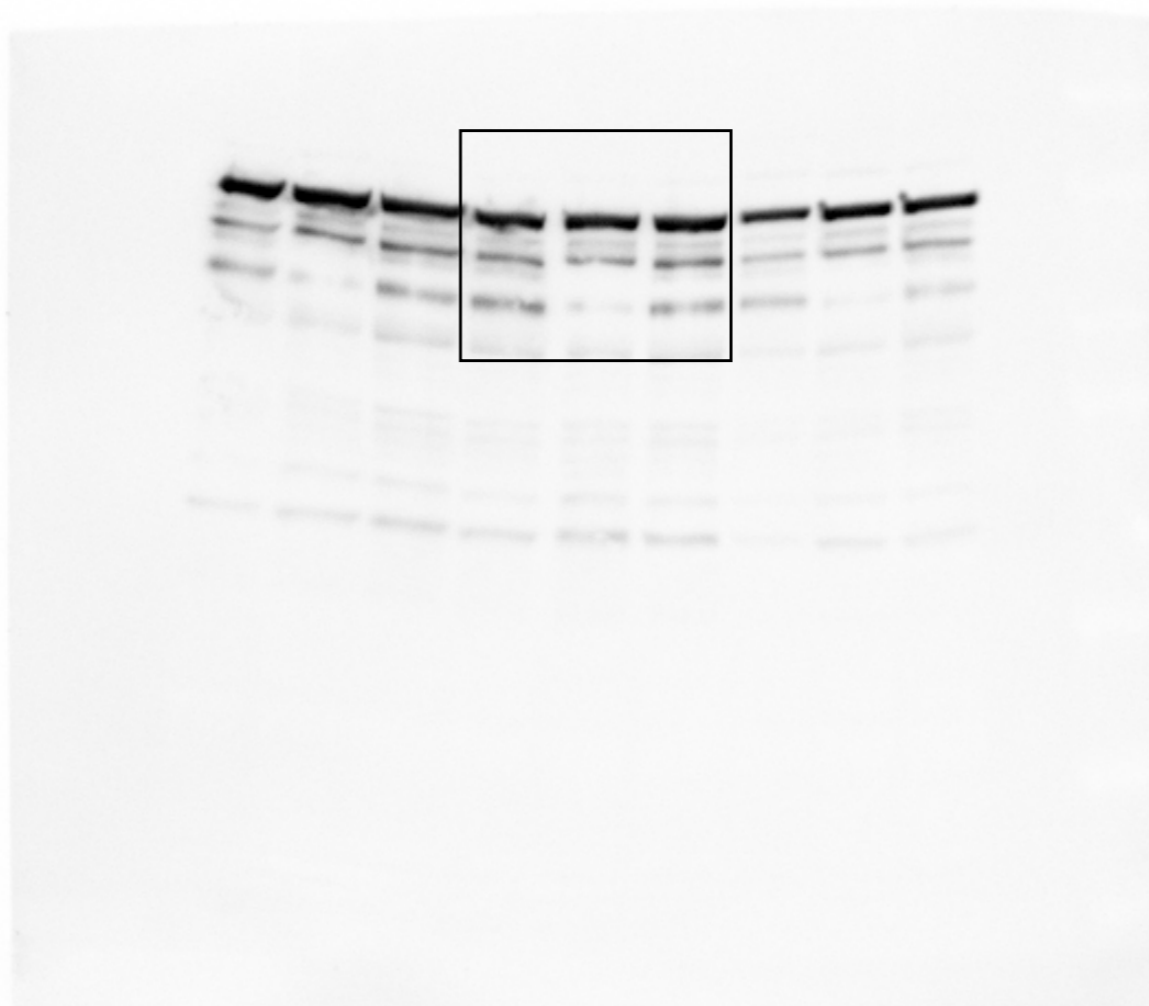

Supplement: Figure 2—figure supplement 2—source data 6. [file elife-74658-fig2-figsupp2-data6.pdf]

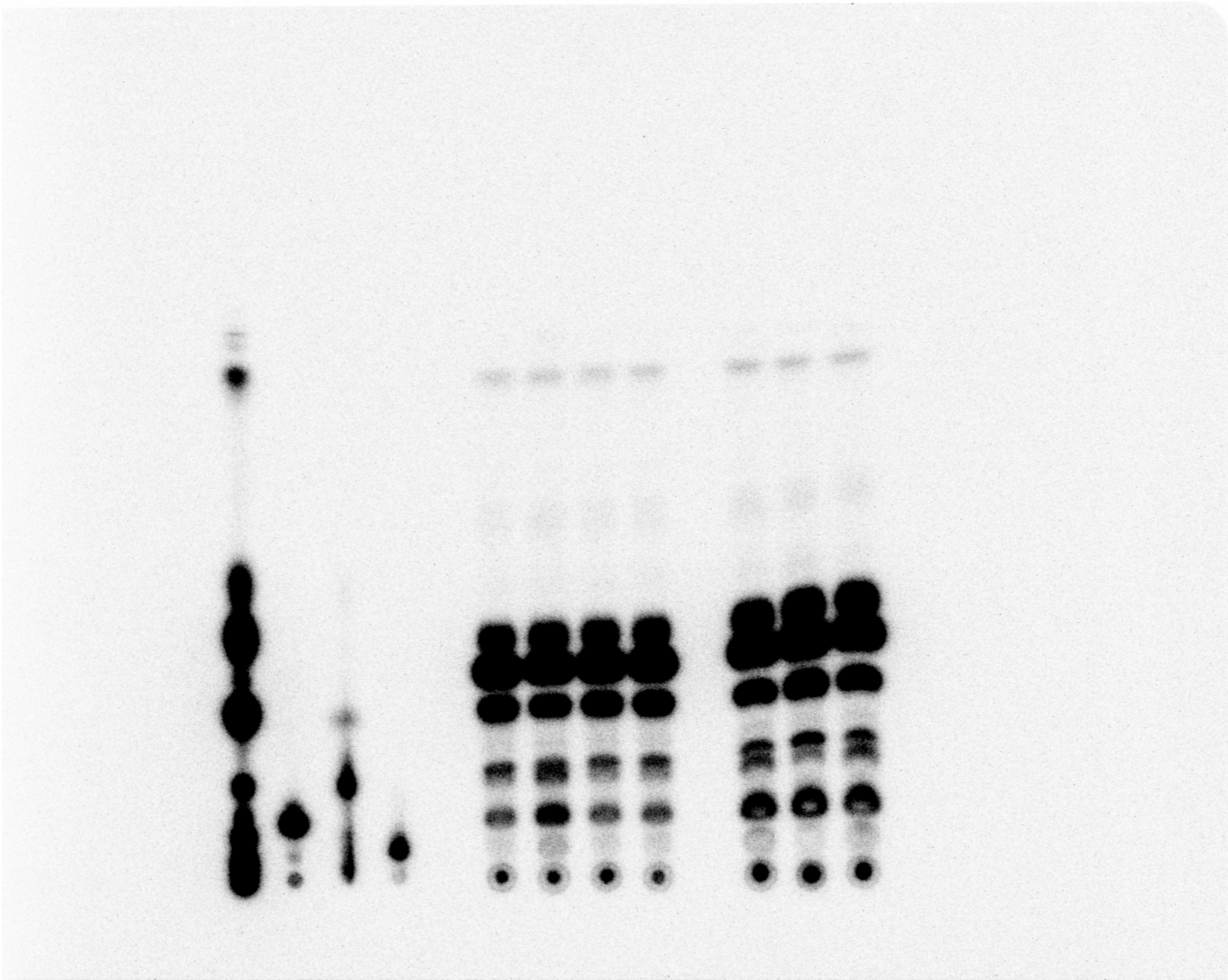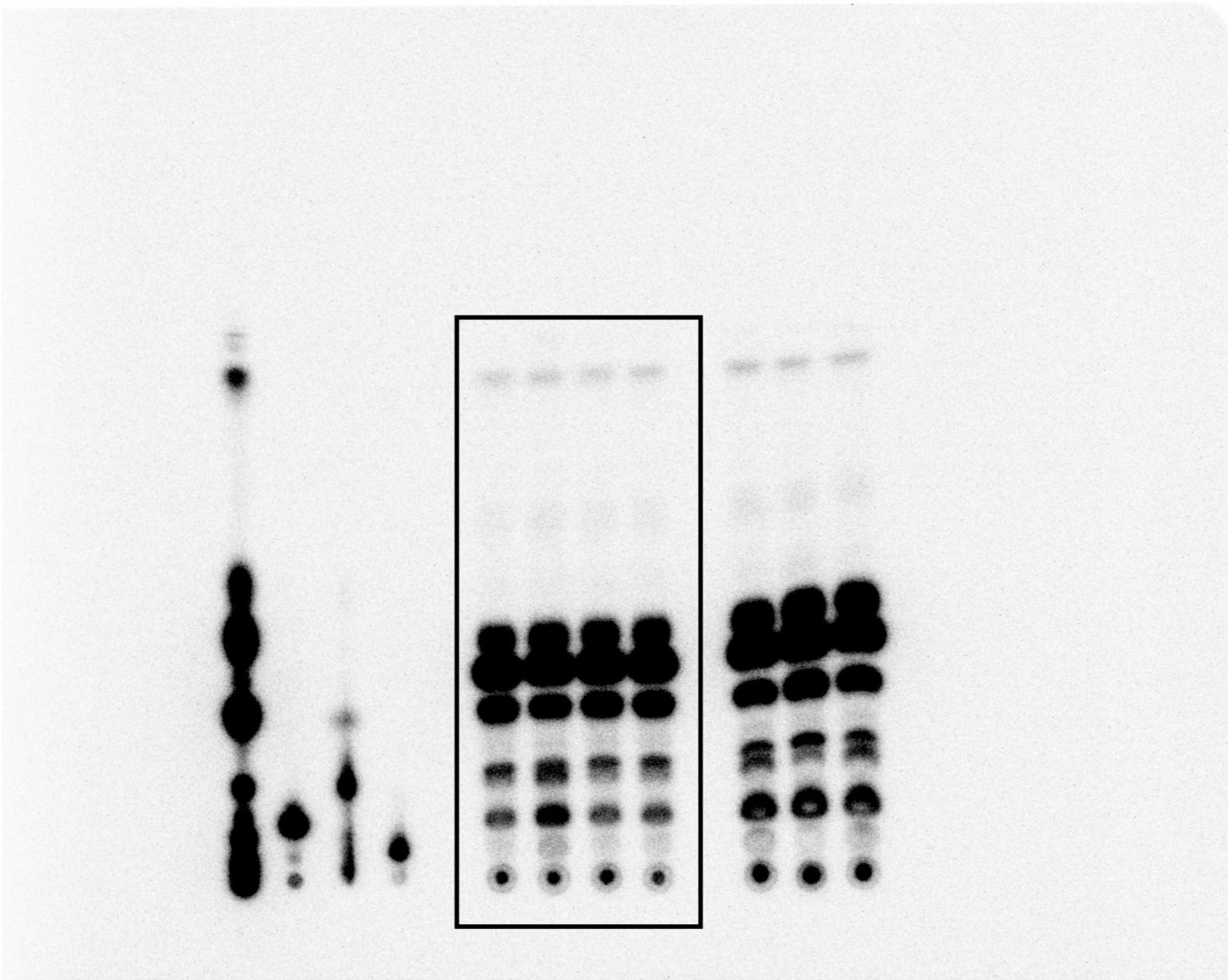

Supplement: Figure 4—source data 1. [file elife-74658-fig4-data1.pdf]

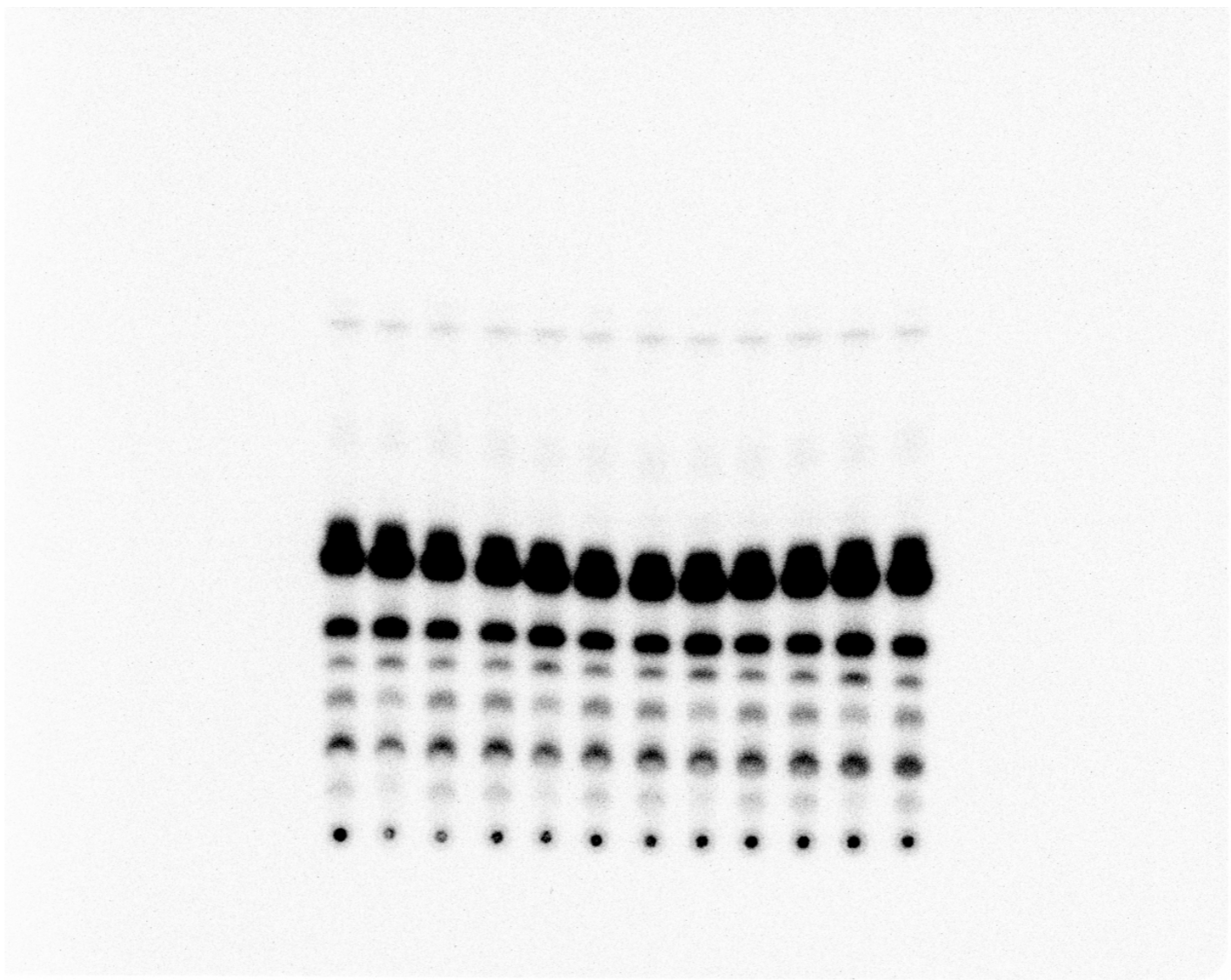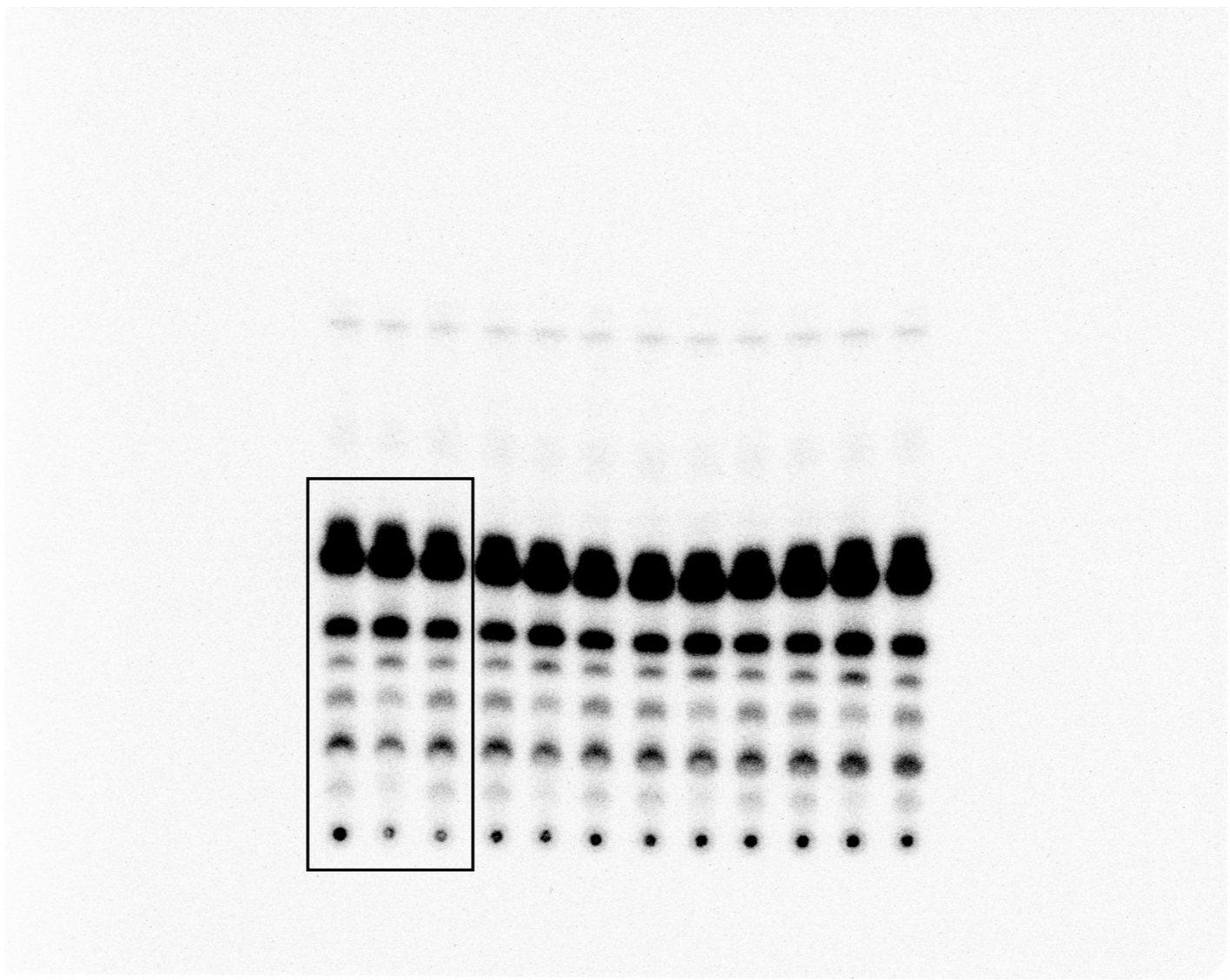

Supplement: Figure 4—source data 2. [file elife-74658-fig4-data2.pdf]
