# Supplementary material for: Discovery of coordinately regulated pathways that provide innate protection against interbacterial antagonism
Source: eLife. 2022 Feb 17;11:e74658. doi: 10.7554/eLife.74658 (PMC8926400; doi:10.7554/eLife.74658)

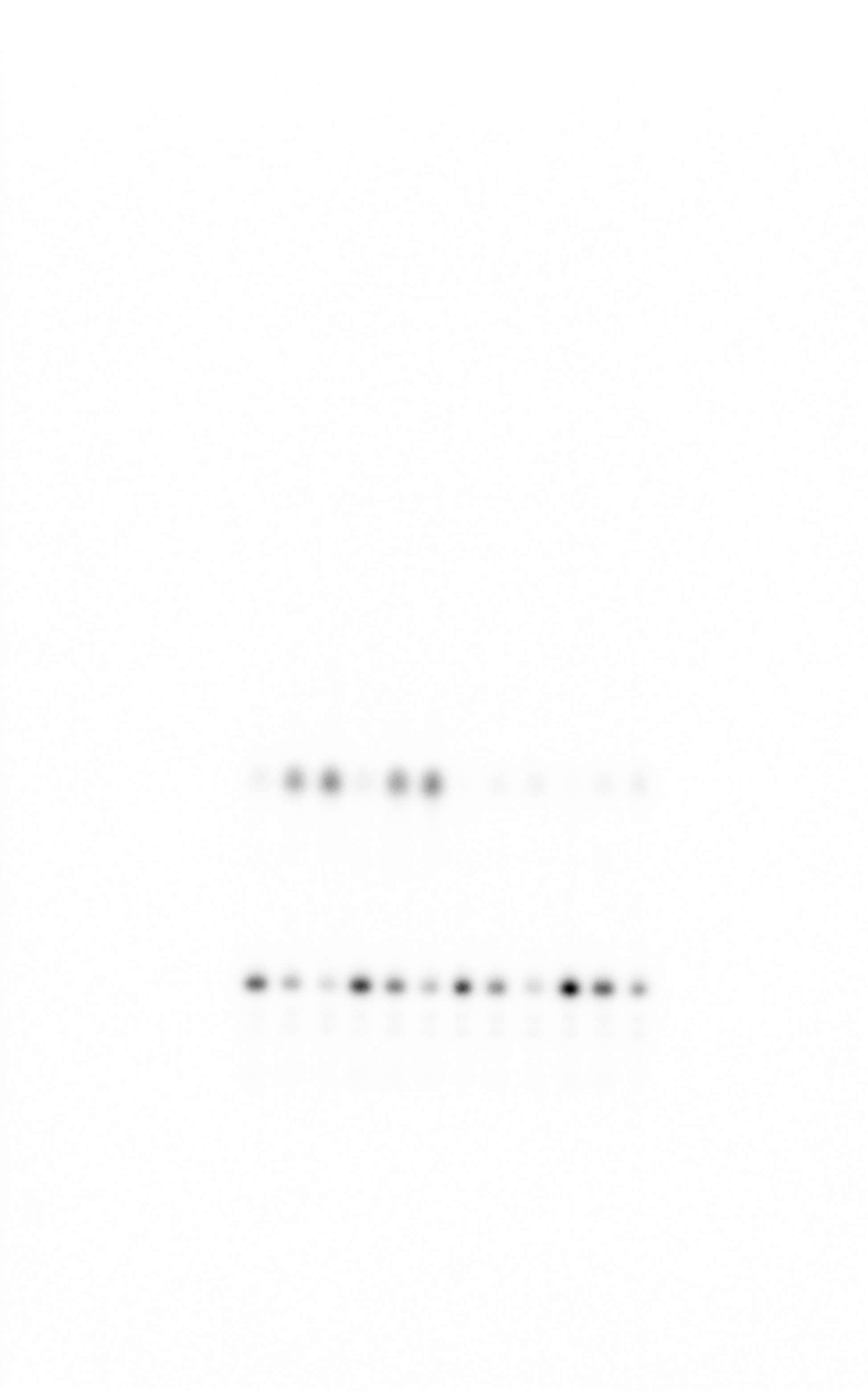

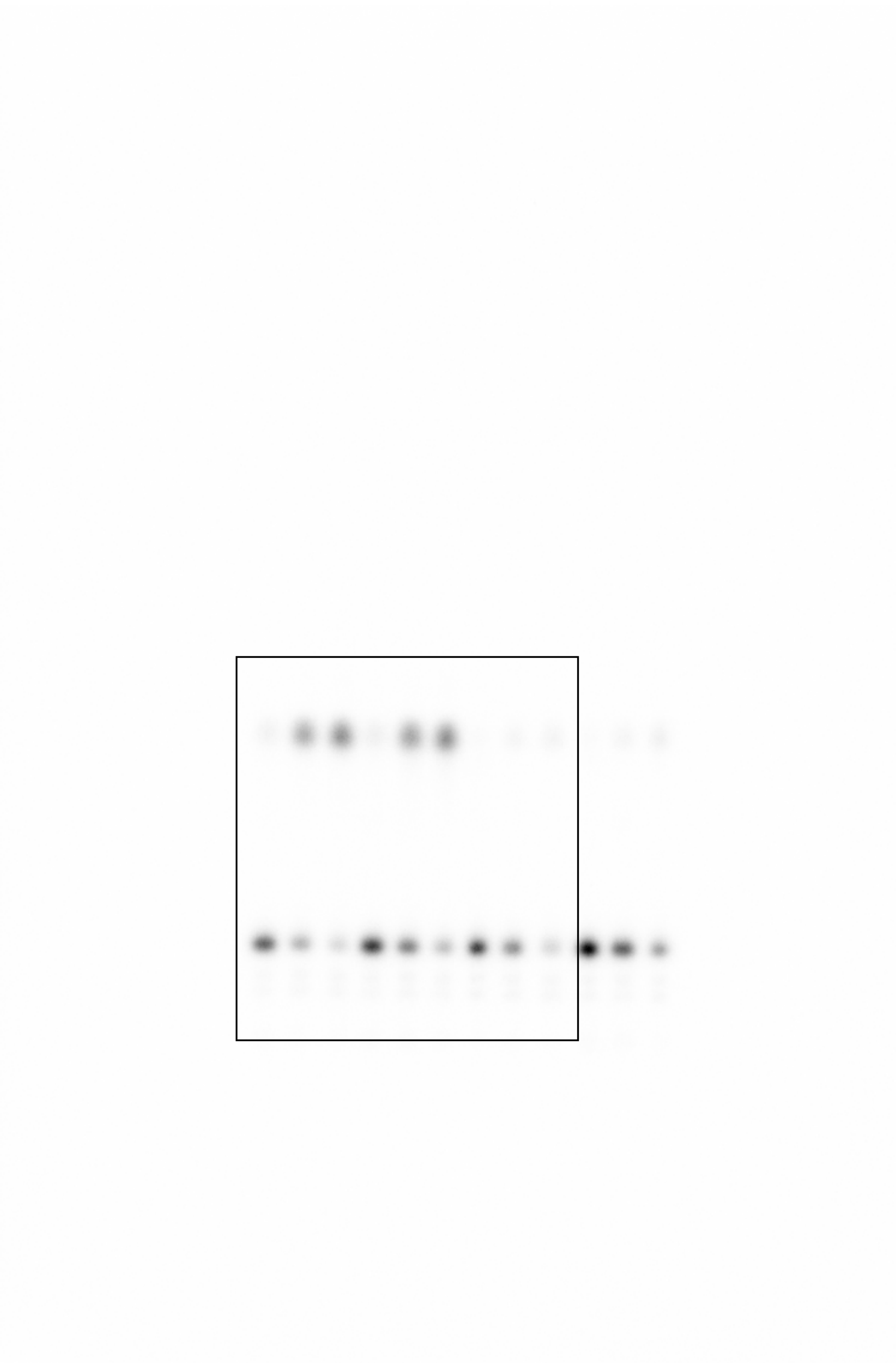

Supplement: Figure 4—source data 3. [file elife-74658-fig4-data3.pdf]
